# Immune cell senescence drives responsiveness to immunotherapy in melanoma

**DOI:** 10.1186/s12943-025-02517-1

**Published:** 2025-12-10

**Authors:** Pavlos Pantelis, Dimitrios Christos Tremoulis, Konstantinos Evangelou, Panagiotis Bakouros, Sophia Magkouta, Orestis A. Ntintas, Dimitris Veroutis, Giorgos Theocharous, Ioannis V. Kostopoulos, Dimitris-Foivos Thanos, Eftychia Chatziioannou, Ioanna A. Anastasiou, Nefeli Lagopati, Dimitrios Valakos, Dimitrios Skaltsas, Oltin Tiberiu Pop, Marie Therese Abdou, Sarantis Gagos, Dimitris Kletsas, Dimitris Thanos, Alexandros J. Stratigos, Martin Röcken, Lukas Flatz, George P. Chrousos, Dimitrios Vlachakis, Ourania E. Tsitsilonis, Russell Petty, Timokratis Karamitros, Vassilis G. Gorgoulis

**Affiliations:** 1 Department of Histology and Embryology, Molecular Carcinogenesis Group, Medical School, National and Kapodistrian University of Athens, Athens, 11527 Greece; 2 Bioinformatics and Applied Genomics Unit, Hellenic Pasteur institute, Athens, 11521 Greece; 3 Department of Biology, Flow Cytometry Unit, National and Kapodistrian University of Athens, Athens, 15701 Greece; 4 Division of Cancer Research, Ninewells Hospital and Medical School, University of Dundee, Dundee, DD19SY UK; 5 Intelligencia Inc, New York, NY 10014 USA; 6 Department of Dermatology, Division of Dermatooncology, University of Tuebingen, Tuebingen, 7207 Germany; 7 First Department of Dermatology-Venereology, National and Kapodistrian University of Athens, Andreas Sygros Hospital, Athens, 161 21 Greece; 8 Laboratory of Biology, Department of Basic Medical Sciences, Medical School, National and Kapodistrian University of Athens, Athens, 11527 Greece; 9 Biomedical Research Foundation, Academy of Athens, Athens, 11527 Greece; 10 Institute of Immunobiology, Kantonsspital St. Gallen, St. Gallen, CH-9007 Switzerland; 11 Laboratory of Cell Proliferation and Ageing, Institute of Biosciences and Applications, National Centre for Scientific Research “Demokritos”, Athens, 15341 Greece; 12 University Research Institute of Maternal and Child Health and Precision Medicine, Medical School, National and Kapodistrian University of Athens, Athens, 11527 Greece; 13 UNESCO Chair On Adolescent Health Care, National and Kapodistrian University of Athens, Athens, 11527 Greece; 14 University Research Institute, Choremeion-Aghia Sophia Children’s Hospital, Athens, 11527 Greece; 15 Department of Biotechnology, Laboratory of Genetics, School of Applied Biology and Biotechnology, Agricultural University of Athens, Athens, 11855 Greece; 16 Algorithms and Bioinformatics Group, Informatics Department, Faculty of Natural, Mathematical & Engineering Sciences, King’s College, Strand Campus, London, WC2R 2LS UK; 17 Faculty Institute for Cancer Sciences, Manchester Academic Health Sciences Centre, University of Manchester, Manchester, M20 4GJ UK

**Keywords:** Immunotherapy, Immune cell senescence, Responders, Non-responders, Melanoma, SeneVick, GLF16, Senoprobe

## Abstract

**Background:**

Immunotherapy has significantly improved cancer treatment. However, it is not effective in all cancer patients, rendering the need to further delineate the differences among responders and non-responders at the molecular and cellular level. Unresponsiveness to immunotherapy has been attributed to dysfunctional immune cell states such as T-cell exhaustion and anergy, whereas the contribution of cellular senescence remains elusive. Herein, we have investigated the role of immune cell senescence in the response to checkpoint inhibitors in melanomas where these immunotherapies are applied as a first line treatment.

**Methods:**

Two senescence detecting complementary approaches were utilized in a case control study we conducted. First, we implemented a senescence molecular signature we developed, termed "SeneVick", retrospectively in a single cell RNA-seq dataset from melanoma patients who received immunotherapy. Prior to this analysis, the signature was extensively validated in a variety of cell/tissue contexts, senescence types and species. Second, cellular senescence was assessed via an established experimental algorithmic approach in circulating immune cells of an analogous melanoma clinical cohort.

**Results:**

Melanoma patients who did not respond to immunotherapy exhibited increased cellular senescence in the CD8 + T-cell, CD4 + T-cell, B-cell (CD19 + /CD20 +) and NK cell compartments compared to responders. This phenomenon was independent of patients’ clinical features (age, sex, melanoma type, stage) and not an outcome of immunotherapy, in contrast to conventional anti-cancer treatments. Interestingly, alterations of cell-to-cell interactions among the immune sub-populations in non-responders compared to responders were identified, supporting, along with cytotoxicity assays, that senescent immune cells display immunosuppressive properties driving defective immune responses and treatment failure.

**Conclusion:**

Overall, our findings provide evidence that cellular senescence within the immune cell compartment of the tumor micro-environment is a potent determinant of the response to immunotherapy and pave the way for strategies targeting it as promising approaches to improve the outcome of such interventions.

**Supplementary Information:**

The online version contains supplementary material available at 10.1186/s12943-025-02517-1.

## Background

Following its initial discovery by Hayflick and Moorhead more than 60 years ago, as "aging at the cellular level", noteworthy advancement has been achieved towards characterizing a cellular stress response mechanism that is distinct from the aging process, termed cellular senescence [1]. Physiologically and on a transient basis, senescence acts as a homeostatic mechanism, limiting the propagation of damaged cells in tissues. In contrast, if senescent cells are not timely eliminated by the immune system, they persist and accumulate, resulting in detrimental outcomes such as age-related pathologies and aging [1].

For many years, a major drawback in the senescence field was the absence of reliable markers to effectively recognize senescent cells [2]. Identification of senescence relied mainly on the Senescence-Associated β-Galactosidase (SA-β-Gal) method, which is applicable only in cell culture and prone to false outcomes [3, 4]. Moreover, other indirect and non-specific senescence markers were commonly applied. Overall, these approaches often resulted in misleading conclusions [5]. In order to bypass these obstacles, we and others recently proposed a senescence detecting algorithm (SDA) that increases the sensitivity and specificity of senescence identification. It also allows for its accurate verification in any kind of biological sample, including formalin-fixed and paraffin-embedded (archival) material [1, 4]. An essential component of SDA is the detection of lipofuscin, a hallmark and a common denominator of all senescent cells [1, 4, 5].

The implementation of SDA retrospectively in clinical samples unveiled that cellular senescence is implicated in various pathologies such as cancer, COVID-19 disease, and giant cell arteritis (GCA), denoting that its role in the pathophysiology of human diseases largely remains encrypted and overlooked [1, 6–8]. Interestingly, in cancer, one of the most common age-related diseases, it has been demonstrated that senescent cells act as a source of tumor recurrence via the senescence-associated secretory phenotype (SASP) and/or the "escape from senescence" phenomenon [1, 4, 9, 10], suggesting its involvement in the clinical outcome of cancer patients [11]. However, implementation of SDA in retrospective analyses will take time to provide results as it is labor-intensive, and in many cases the material is limited or even exhausted. Moreover, given the complex and largely heterogeneous nature of the senescence phenotype, tools that facilitate towards precise senescence identification are necessary to further elucidate its role in the pathophysiology and clinical course of human pathologies, such as cancer [12, 13]. These conundrums along with the unexplored for senescence abundant single cell RNA-seq (scRNA-seq) data available databases and the important drawbacks of existing senescence detection pipelines led us to develop a molecular signature, from now on termed "SeneVick", that could complement SDA in identifying senescence accurately [14, 15]. As demonstrated, following extensive validation, SeneVick proved a highly efficient tool in demarcating non-senescent from the senescent state. Immunotherapy exemplified by checkpoint inhibitors has drastically influenced cancer therapy in the last decades [16]. These treatments aim to increase the efficacy of immune cells against the tumor. However, current cancer immunotherapies are not effective in all patients [17]. Cancer-induced immunodeficiency is an important determinant of the response to such interventions, however, the molecular mechanisms though governing these processes remain to a large extent, unresolved [18]. Thus, an interesting matter that emerges regards the biological events that distinguish Responders (Rs) from Non-Responders (NRs) to immunotherapy. Dysfunction of the immune cell compartment within the tumor microenvironment (TME), as an outcome of immune cell exhaustion or anergy, has been proposed while the involvement of immune cell senescence remains uncharted [5]. Herein, we address this topic by implementing in a complementary manner experimental and *in silico *approaches, signifying that NRs to immunotherapy melanoma patients exhibit increased immune cell senescence in CD4+ and CD8+ T-cell, B-cell (CD19+/CD20+) and natural killer (NK) cell populations compared to Rs. In line with these findings, we showed for the first time that senescent T-cells demonstrate dysfunctional properties favoring immune suppression and resistance to immunotherapy.

## Materials and methods

### Experimental planning

Prior to addressing the main question of our study, whether immune cell senescence drives responsiveness to immunotherapy in melanoma patients (*"In silico* datasets and *ex vivo *melanoma setting"), we extensively validated our molecular senescence signature, SeneVick, in *in silico* (Sections: "*In silico *setting", "Analysis of the senescence signature SeneVick" and "Gene set enrichment analysis") and experimental senescence models (Sections: "*In vitro *setting", "Senescence assessment", "Telomere analysis", "Transcriptomics" and "Cytotoxicity assay").

### Senescence models

#### * In silico* setting

The analysis of the senescence control datasets, which included scRNA-seq data from mice of different age groups and human fibroblasts (WI-38) [19, 20], was conducted as outlined below: The scRNA-seq data (GSE132042 and GSE226225) for the mice cohort and human fibroblasts (WI-38), respectively, were downloaded from Gene Expression Omnibus (GEO). Particularly, the first control dataset [19] (GSE132042) contained scRNA-seq data from various tissues of mice belonging to six age groups, from 1 to 30 months. The second dataset (GSE226225) includes scRNA-seq data from human fibroblasts undergoing radiation- or therapy-induced senescence following Etoposide (ETO) treatment [20]. Cells with less than 1000 detected genes were omitted from the analysis. The gene counts were decontaminated using DecontX [21] (v1.0.0). Seurat (v5.0.1) was used for the main part of the analysis. The quality control steps included filtering of cells that had a mean expression > 2^2.5–1 of selected housekeeping genes (Table S1) and the mitochondrial counts were removed. The cells were integrated with the fastMNN function (only in the fibroblast dataset) and clustered using a clustering resolution of 0.3 and 40 principal components.

In the mouse senescence control dataset, which contained a publicly available single-cell transcriptomic atlas that was extracted across the lifespan of *Mus musculus* [19] linear models were used to determine whether there is a linear relationship between the timepoints and the senescence signature score. A Wilcoxon test was conducted per cell type between those two groups, in order to determine senescence score differences.

The senescence enrichment score of each signature [14, 22–24] (SeneVick, SenMayo and Fridman) in human fibroblasts derived from the human senescence control dataset [20] (GSE226225) was compared via Wilcoxon test. Furthermore, we compared the enrichment levels of the aforementioned signatures in the human fibroblasts, in which the induction of senescence was accomplished with different senescence inducers (IR-irradiation, ETO treatment), and significance was also assessed via Wilcoxon Test. Segmented linear regression (segmented R package, v.2.1.3) was used in order to model the increase of senescence enrichment scores of the different senescence signatures across several timepoints, following ETO treatment.

####  *In vitro *setting

Human diploid WI-38 fibroblasts (purchased with from ATCC, CCL 75) and Primary Human Fibroblasts (kindly provided by the Laboratory of Cell Proliferation and Ageing, NCSR "Demokritos" ) of the three different age groups (7-, 35-, 75- years) were cultured in Dulbecco’s modified Eagle medium (DMEM, Biowest, L0104) supplemented with 10% FBS and 1% antibiotics. Cell cultures were maintained in an incubator at 37° C and 5% CO2. For ETO-induced senescence, human diploid WI-38 fibroblasts were treated with 50 μM ETO (for six days), then cultured in regular medium without ETO-containing medium for four additional days. In the time course experiments, cells were collected at 0 (untreated), 1, 2, 4, 7, and 10 days after ETO treatment.

Human Peripheral blood mononuclear cells (PBMCs) from healthy donors were isolated using Ficoll (1.077 g/ml) following standard procedures [25]. Cells were cultured in RPMI medium (Roswell Park Memorial Institute 1640 Medium) supplemented with 5% Cell-Vive™ T-NK Xeno-Free Serum Substitute (Biolegend) and 200 IU/ml hIL-2. T-cells were subsequently isolated using the MojoSort™ Human CD3 T-Cell Isolation Kit (Biolegend) and then they were treated with vehicle (PBS for non-senescent control cells) or cisplatin (100 μM) for 48 h to induce ROS-mediated senescence [26, 27] (senescent T-cells).

### Analysis of the senescence signature SeneVick

#### Gene ontology-based functional annotation

Gene Ontology (GO) enrichment analysis was performed to identify significantly overrepresented biological themes in SeneVick among its genes. GO is a hierarchically structured vocabulary encompassing three domains: Biological Process (BP), Molecular Function (MF), and Cellular Component (CC). Each domain captures different facets of gene function, allowing comprehensive annotation across cellular contexts. GO annotations and enrichment testing were conducted using g: Profiler [28] and Database for Annotation, Visualization and Integrated Discovery (DAVID v.6,8) [29] Knowledgebase v2023q4 as updated quarterly. Analyses were carried out using the Homo sapiens reference background (Ensembl GRCh38), and significance was determined using a hypergeometric test followed by Benjamini–Hochberg false discovery rate (FDR) correction. Only GO terms with FDR-adjusted p-values < 0.05 were statistically significant. To complement the GO-based analysis, pathway annotations were retrieved from Kyoto Encyclopedia of Genes and Genomes (KEGG), Reactome, and BioCartadatabases. These included canonical signaling pathways relevant to senescence, such as the p53/p21^WAF1/CIP1^ axis, NF-κB activation, mitochondrial metabolism, SASP regulation, and DNA damage response (DDR). Each gene was mapped to one or more functional categories based on GO and pathway term enrichment.

#### Construction of functional association matrix

A binary gene-function matrix was constructed in which rows represented individual genes and columns represented significantly enriched GO terms and pathways. Each matrix entry was coded as "1" if the gene was associated with a given term, and "0" otherwise. To reduce dimensionality and remove redundancy due to overlapping terms, Principal Component Analysis (PCA) was applied, retaining components explaining > 90% of the total variance. To delineate distinct biological modules within the senescence signature, unsupervised clustering was performed on the reduced gene-function matrix. Two complementary approaches were applied:

1. K-means clustering [30] was implemented using the Euclidean distance metric. The optimal number of clusters (k) was selected by evaluating the elbow plot and silhouette score across a range of k -values. This method identified non-overlapping clusters of genes sharing similar functional annotation profiles.
2. Agglomerative hierarchical clustering was performed using Ward’s linkage method, producing a dendrogram to evaluate hierarchical relationships among functional groups. Final clusters were defined by cutting the dendrogram at a level that maximized within-cluster similarity while preserving between-cluster separation.

Genes associated with multiple terms were assigned to the cluster in which they showed the highest cumulative enrichment score. In cases of ambiguity, gene membership was resolved based on semantic similarity scoring, calculated using the GOSemSim R package. This enabled biologically meaningful classification based on ontological proximity to core senescence processes. All analyses were conducted in R version 4.3.0 and Python 3.10, using the packages clusterProfiler, factoextra, GOSemSim, scikit-learn, and SciPy. GO and pathway databases were accessed in June 2025 to ensure current annotation status.

### Senescence assessment

Senescence assessment was performed in cells following double staining with the senescence detecting reagent GLF16 that we generated along with related senescence markers, and in tissues by applying the senoprobes GL13 [31] and GLF16, according to the SDA [3, 24, 32], as follows:

#### GLF16 staining/Immunofluorescence

Primary human skin fibroblasts of three different age groups (7-, 35-, and 75- years) or human diploid WI-38 fibroblasts were seeded (2 × 10^5^ cells/well) on coverslips (12-mm diameter). The latter were subsequently treated with Etoposide for senescence induction [20]. In both cases, coverslips were subsequently removed; cells were washed, fixed (4% PFA/PBS, 10 min, 4 °C) and permeabilized (Triton 0.3%/PBS 15 min). Blocking of non-specific epitopes was performed using sheep serum (dilution 1/40, S22, Merck Millipore). Cells were subsequently stained for lipofuscin using GLF16 for 10 min (70 mg/ml) avoiding light exposure as previously described [24]. Coverslips were washed 3 times for 10 min each with GLF16 diluent (2.5% DMSO/2.5% Tween-20/95% PBS). Then, cells were incubated with anti-p16^ΙΝΚ4Α^ (16D5, QR Labs) or -p21^WAF1/CIP1^ (1947S, Cell Signaling) antibodies for 1 h at room temperature (RT), followed by application of appropriate secondary antibodies (for 1 h in RT). Nuclei were finally visualized by DAPI. Cells were washed (30s with dH_2_O) and coverslips were mounted onto slides for microscopy. T-cells were isolated from PBMCs and treated as described in section "*In vitro* setting". Cytospins of 1 × 10^5^ cells were prepared using the cytocentrifuge (400 g, 5 min) and stained with GLF16 according to the SDA mentioned in 2.4.1.

In the case of tissues, 4-μm-thick sections of formalin-fixed and paraffin-embedded tissues (FFPE) were obtained, de-paraffinized and hydrated. Antigen retrieval was performed by immersing samples in 10 mM of citric acid buffer (pH 6.0) in a steamer for 15 min. Tissue samples were cooled down and washed with PBS. Blocking of non-specific binding for the epitopes was done by applying normal goat serum for 1 h at room temperature (dilution 1:40, Abcam, Cambridge, UK ab138478). The samples were incubated with the following primary antibodies overnight at 4^◦^C: CD4 (Ready to use, M7310, Dako), CD8 (1:20, 144B, Dako) and CD20 (1:150, 250586, Abbiotech). Positive cells were visualized using secondary goat anti-mouse (Abcam, ab6785, polyclonal) and goat anti-rabbit immunoglobulin G and heavy and light chains (IgG H&L antibody, Alexa Fluor 488; 1:500; Abcam, ab150077, polyclonal) for 1 h. Upon staining with primary and secondary antibodies, tissue sections were stained for lipofuscin applying GLF16 for 10 min (70 μg/ml) in the dark. Excess compound was removed by washing three times with the GLF16 diluent (2.5% DMSO/2.5% Tween 20/95% PBS). Nuclei were finally visualized by DAPI staining. The samples were washed (30 s with dH_2_O), and coverslips were mounted onto slides for microscopy. Samples were imaged using a Leica TCS-SP8 confocal microscope.

#### GL13 staining

FFPE sections from melanomas were de-paraffinized and hydrated. Subsequently, antigen retrieval was carried out as described in section "Senescence assessment" and after blocking of non-specific binding sites with goat serum (Abcam ab1388478, in 1:40) and Hydrogen Peroxide (H_2_O_2_), (Dako REAL EnVision Detection System kit Cat.no: K5007, Santa Clara, CA, USA) the tissues were incubated sequentially in 50% and 70% ethanol for 5 min each, respectively. Following application of GL13 on each tissue, the samples were incubated at 37 °C for 10 min. At the end of this step, the samples were washed with 50% ethanol for 2–3 min, with PBS and then Triton-X 0.3%/PBS was applied for 5 min in order to remove any reagent precipitates. Tissues were washed again with PBS and anti-biotin antibody (in dilution 1:300, Hyb-8, ab201341, Abcam, Cambridge, UK) was applied and incubated for 1 h at RT. The mean percentage of GL13-positive cells was assessed from ≥ 5 high-power fields (Objective 40×) per sample using a ZEISS Axiolab5 optical microscope.

#### Immunocytochemistry—Immunohistochemistry

Cells from each cell line were seeded on coverslips as mentioned above. For the Immunocytochemistry (ICC), the cells were permeabilized using Triton-X 0.3%/PBS for 15 min at RT, followed by the blocking of non-specific binding sites with goat serum [33] (Abcam ab138478, in 1:40) for 1 h at RT and H_2_O_2_ for 18 min. Cells were then incubated with Ki67 (Cat.no: ab16667, dilution 1:250, SP-6, Abcam, Cambridge, UK) for 1 h at RT. Positive cells were visualized using the Dako REAL EnVision Detection System kit (Cat.no: K5007, Santa Clara, CA, USA) according to the manufacturer’s instructions using 3,3′-Diaminobenzidine (DAB) (brown color). Coverslips were counterstained with hematoxylin, sealed and observed under a ZEISS Axiolab5 (Munich, Germany) optical microscope with 20× or 40× objectives.

Regarding the FFPE material (Section: "GLF16/Immunofluoresence"), sections were incubated with anti-CD4, anti-CD8 and anti-CD20 antibodies, overnight at 4^◦^C, respectively: CD4 (Ready to use, M7310, Dako), CD8 (1:20, 144B, Dako) and CD20 (1:150, 250586, Abbiotech). Positive cells were visualized using the Dako REAL EnVision Detection System kit (Cat.no: K5007, Santa Clara, CA, USA) according to the manufacturer’s instructions using 3,3-Diaminobenzidine (DAB). Sections were counter-stained with hematoxylin and observed using a ZEISS Axiolab 5 optical microscope with a 20× objective, 25 µm scale bar.

### Telomere analysis

#### Telomere length measurement

Relative telomere length determination (T/S) refers to the ratio of telomere (T) hexamer repeat sequence TTAGGG signal, to autosomal single copy gene (S) signal. To assess this, cells were collected and frozen at −80 °C until all samples were ready for simultaneous DNA extraction and analysis. Genomic DNA was extracted using the T3010 Monarch Spin gDNA Extraction Kit (T3010, New England Biolabs). Telomere ("T") and single copy gene (human albumin, "S") lengths were measured via real-time PCR (Roche LC480, Roche Diagnostics Corporation, Indianapolis, IN). Samples were loaded on 96-well plates and run in triplicate. Repeated measures of the T/S ratio in the same DNA sample gave the lowest variability when the sample well position for T-PCR on the first plate matched its well position for S-PCR on the second plate. When one sample's duplicate T/S values differed by greater than 7%, the sample was run a third time, and the two closest values were averaged to give the final result. This ratio was subsequently normalized by control DNA samples to yield relative standardized T/S ratios proportional to average telomere length. A 5-point standard curve (made of pooled reference DNA samples (100 to 6.25 ng/uL) and randomly located internal QC sample replicates (*n* = 5), were utilized as calibrator samples, to guide analysis and indicate overall quality of assay performance. Additionally, a non-telomeric control was added to random well locations to provide a unique fingerprint for each plate. The primers (100μΜ, Integrated DNA Technologies Coralville, IA) used were the following:

i. telomeric assay:
  TelG [5’-ACACTAA GGTTT GGGTT TGGGTT TGGGTTT GGGTT AGTGT-3’].
  TelC [3’-T GTTAGG TATC CCTA TCCCTAT CCC TATCC CTA TCCC TAACA-5’]
ii. single-copy gene (Albumin) assay:
  AlbU [5’-C GGCGG CGG GCGG CGCGG GCTG GGCGG AAATG CTGCACA GAAT CCTTG-3’]
  AlbD [5’-G CCCGG CCC GCCGC GCCC GTCC CGCCG GAAAA GCAT GGTC GCCTGTT-3’] PCR was performed using 20uL reaction volumes consisting of: 10 uL of 2X Luna® Universal qPCR Master Mix (NEB, US), 7.0 uL of Molecular Biology Grade (MBG) Water, and 0.5 uL of 1 µM primers mix. Thermal cycling was performed on a LightCycler 480 (Roche) where PCR conditions were (i) T (telomeric) PCR: 95˚C hold for 5 min, denature at 98˚C for 15 s, anneal at 54˚C for 2 min, with fluorescence data collection, 35 cycles and (ii) S (single-copy gene, Alb) PCR: 98˚C hold for 5 min, denature at 98˚C for 15 s, anneal at 58˚C for 1 min, with fluorescence data collection, 43 cycles. Ct-values of triplicates were averaged, if meeting a CV threshold of less than 2%. The telomere (T) concentration was divided by the albumin (Alb) concentration (S) to yield a raw T/S ratio. Raw T/S ratios were subsequently normalized by average internal QC calibrator samples within the same plate set. Z-scores were calculated to adjust RTL in case differences in dynamic range are introduced by systematic differences between batches.

#### Telomeric peptide nucleic acid (PNA) FISH

Telomeric PNA Fluorescence* In Situ* Hybridization (FISH) was held according to the latest established protocols [34]. The primary skin human fibroblast cells of the three different age groups (7-, 35- and 75- years) were cultured in a confluency of 60–80% and they were split at 48–72 h before harvesting for metaphase chromosomes. Cell pellets were fixed with methanol and acetic acid and dropped on wet slides for overnight incubation. Cells were subsequently re-hydrated using PBS (15 min, RT) and subsequently incubated with RNase A (100 μg/μl, Merck KGaA, Darmstadt, Germany) for 1 h at 37 °C. Chromosome preparations were fixed in 3.7% formaldehyde (2 min) and washed with TBS (twice, 5 min each). Chromosome preparations were digested with pepsin (1 mg/ml, in 10 mM HCL, pH 2) at 37 °C for 10 min and then washed twice with TBS and finally dehydrated by serial incubations in 70, 85, and 96% cold ethanol and air‐dried. Telomere‐specific hybridizations were accomplished employing Cy3‐labeled (TTAGGG)3 and FITC‐labeled (CCCTAA) 3 PNA probes (BioSynthesis, Lewisville, TX). After two consecutive washing steps, one in PBS and one in Wash solution (0.1 M Tris–HCL, 0.15 M NaCl, for 5 min. Subsequently, 10 μl of hybridization mixture comprising of 0.2–0.8 μM PNA telomeric probes, 70% formamide, and 10 mM Tris, pH 7.2 (Cytocell, Oxford Gene Technology, UK), was applied to the marked area of the slide. The latter underwent heating at 80 °C for 5 min during the denaturing FISH protocol, whereas this step was excluded in the non-denaturing FISH procedure. Slides from both denaturing and non-denaturing FISH procedures were incubated overnight at 37 °C in a humid environment. On the following day, slides were sequentially washed: once in PBS for 15 min, once in 0.5× Saline Sodium Citrate buffer (SSC) containing 0.1% SDS at 72 °C for 2 min, once in 2× SSC (pH 7) supplemented with 0.05% Tween-20 at room temperature for 30 min, and twice more in PBS for 15 min each. Preparations were then counterstained and mounted with Vectashield containing DAPI (Vector Laboratories Inc). Images were captured under an Axion Imager Z1 Zeiss fluorescence microscope (63× objective) and analyzed using MetaSystems Isis software. The signal of the centromere of chromosome 2 functioned as the internal reference control. Human centromere 2‐specific PNA probes labeled with Cy3 or FITC were provided from DAKO Cytomation (Glostrup, Denmark).

### Transcriptomics and analysis

#### Transcriptomics

Primary human Fibroblasts of 7-, 35-, and 75-years old donors and PBMCs from Rs and NRs patients isolated as described in section "*In vitro *setting" were seeded onto 10-cm cell culture plates (70% confluency). Cells were collected and total mRNA was extracted using the NucleoSpin RNA mini kit (Macherey–Nagel, Germany). RNASeq libraries were prepared with the NEBNext ultra II directional RNASeq kit (Reverse strand specificity) and single end sequenced at 101 bp length with the Illumina NovaSeq 6000 platform, in the Greek Genome Center of BRFAA.

Raw data were mapped to the human genome (version GRCh38/hg38) using STAR [35] aligner. Samtools [36] were used for data filtering and file format conversion, while the HT-seq count algorithm [37] was used to assign aligned reads to exons using the following command line ‘‘htseq-counts non intersection–nonempty’’. Normalization of reads and removal of unwanted variation was performed with RUVseq [38]. Differentially expressed genes were assessed using the DESeq2 R package [39] and the significant genes were characterized by log2 fold change cut-off of 0.5 and *p*-value less than 0.05. Gene ontology and pathway analysis was accomplished using the DAVID software [40]. Only pathways and biological processes with *p*-value less than 0.05 were characterized as significantly enriched. Heatmaps representing the significant differentially expressed genes and the most significant genes where SeneVick was found enriched were constructed with R package Shiny [41], where hierarchical clustering was performed, with linkage method ‘average’.

#### Gene set enrichment analysis

Gene Set Enrichment Analysis [42] (GSEA) was used in order to determine whether SeneVick is enriched in senescent samples. More specifically, the signature’s genes that are expected to be upregulated were tested for enrichment separately from those expected to be downregulated. Age in years was treated as a continuous variable and Pearson correlation was used to determine the enrichment or depletion of the signature’s genes across age.

### Cytotoxicity assay

Human PBMCs from healthy donors were isolated, cultured and the senescence induction was held as referred in section "*In vitro *setting". T-cells were activated using CD3/CD28 activation beads (Biolegend,) (1:1 cell-to-bead ratio) for 3 days. Melanoma tumor cells (A375) were loaded onto U-bottom 96-well plates at a density of 2 × 10^4^ cells/well. Activated T-cells were subsequently added at a 0:1 (no T-cells) 1:1, 10:1 and 25:1 T-cell: tumor cell ratio and co-cultures were incubated for 24 h. Lymphocytes were removed by PBS washing and viable tumor cell numbers were determined by MTS 3-(4,5-dimethylthiazol-2-yl)−5-(3-carboxymethoxyphenyl)−2-(4-sulfophenyl)−2H-tetrazolium, inner salt).

### Melanoma patients

#### *In silico datasets*

##### Integration of databases

The scRNA-seq data [43, 44] for the melanoma cohort (Table S2) were processed as described above in section "*In silico *setting", with the additional steps of a) the conversion of the counts to TPM, log transformation and b) the use of a minimum mean housekeeping expression threshold of log2(TPM + 1) > 2.5. After the quality control steps, the cells were annotated using SingleR (v2.4.1) while using as a reference, a dataset comprising 300.000 immune cells [45, 46]. The response status of the melanoma patients in the *in silico* datasets was assessed according to the Response Evaluation Criteria in Solid Tumours (RECIST) [47].

The dataset was then split into broad cell types and comparisons were made between Rs and NRs. For each cell type, the dataset was integrated using the fastMNN function and clustered using a clustering resolution of 0.3 and 40 principal components. Ucell [48] (v2.6.2) was used to calculate the senescence score, for each of the three aforementioned signatures (SeneVick, SenMayo and Fridman), as well as the T-cell anergy and exhaustion scores. Next, in the cases where cell type contained clusters exhibiting low senescence scores, and high exhaustion scores, these clusters were excluded. A Wilcoxon test was conducted between the Rs and NRs cells, in order to determine senescence score differences. The scRNA data obtained from patients prior to immunotherapy administration were analyzed using the same method. Of note the only difference was noted in the fact that there wasn’t an need for integration of the data and that in cases where the low senescence—high exhaustion cells were not forming a separate cluster such as in CD4 + T-cells, we removed all the cells which had a senescence score below the 20th percentile and simultaneously an exhaustion score above the 80th percentile and used a clustering resolution of 0.5.

##### SeneVick’s cut-off determination to predict immunotherapy response

To assess whether SeneVick could predict the response to immunotherapy in our dataset, we defined a per-patient, senescence index based on our signature:

$$\text{Sene Vick Index}=\frac{\frac{\text{High senescence cells}}{\text{Total cells}}+\text{ps}}{\frac{\text{Low senescence cells}}{\text{Total cells}}+\text{ps}}$$

where ps = 0.01 is a pseudocount to avoid division by zero. The SeneVick index was calculated for each patient with more than 10 cells per cell type (CD4^+^ T-cells and CD8^+^ T-cells). To this purpose, we generated two thresholds: The upper one, above which cells are considered highly senescent, and the bottom one, below which cells are considered marginally senescent. In order to avoid bias in our statistical methodology and since each cell type would be characterized by different optimal thresholds, we ran an optimization analysis. In this analysis, we tested all upper percentiles of senescence enrichment (between 55–95) and all lower percentiles of senescence enrichment (between 5 and 45) with a step size of 5 with the resulting patient indices for each threshold pair evaluated via Receiver operating characteristic (ROC) analysis. The optimal threshold pair was determined as the one that maximized the area under the curve (AUC) score, while retaining the vast majority of patient-derived cells from the *in silico* datasets [43, 44]. The latter was accomplished using Youden’s index [49], which allows for the optimal discrimination of Rs and NRs. All in all, the above methodological framework constitutes a novel approach that is recommended to be followed for effective senescence cut-off determination in the dataset of interest.

##### Cell communication analysis

In order to infer the cell–cell communication between the immune cells the CellChat R (v2.2.0) package was used [50]. Cells were grouped based on their cell type and response status, and the minimum number of cells required per group for the analysis was set to 50. Subsequently, the differences between Rs and NRs Ligand-Receptor pair communication probabilities in CD8 + T-cells, CD4 + T-cells, NK cells and B-cells (CD19 +/CD20 +) were identified.

#### *Εx vivo* melanoma setting

Twenty-four (24) melanoma patients that received single or combinatorial immune checkpoint inhibitors, as first line therapy after entering stage IV, were analyzed. Patient and clinical characteristics are depicted in Table S3. All patients included in this study gave their written consent and the study was approved by the local ethical review board (project ID: Ethikkommission Ostschweiz, EKOS 16/079). Patient’s response was assessed using RECIST version 1.1 [47], approximately 3 months after initiation of therapy and at 3-month intervals thereafter. The Best Overall Response (BOR), defined as the best response recorded from the start treatment initiation until disease progression was documented. Based on BOR, patients were categorized as responders [47] (Rs, complete or partial response) or non-responders (NR, stable disease or progressive disease).

##### Senescence assessment in PBMCs

Peripheral blood mononuclear cells (PBMCs) from the melanoma patients were obtained following established procedures using Ficoll as previously described [25]. PBMCs were thawed, washed twice with PBS containing 0.5% heat-inactivated fetal bovine serum (PAN-Biotech GmbH; staining buffer), and resuspended in staining buffer. Cell viability and concentration were assessed microscopically using a hemocytometer (Neubauer chamber) and Trypan blue staining (Corning®, NY, USA). Cells were set at a final concentration of 5 × 10^6^/ml and 100 μl of them were then transferred to a 5 ml round-bottom polystyrene Flow Cytometry Analysis (FACS) tube (BD Biosciences, NJ, USA). Cells were labelled with BD Horizon™ Fixable Viability Stain 570 (BD Biosciences, NJ, USA) for 20 min at 4 °C in the dark and washed twice with staining buffer, before adding the master mix of 15 fluorochrome-conjugated monoclonal antibodies (Table S4) targeting surface antigens (BD Biosciences, NJ, USA; Biolegend Inc., CA, USA). To minimize non-specific binding, 10 μl of BD Pharmingen™ MonoBlock™ buffer (BD Biosciences, NJ, USA) was added together with the antibody master mix, and cells were incubated for 30 min at 4 °C in the dark. Further, cells were fixed and permeabilized using the BD Pharmingen™ Human FoxP3 Buffer Set (BD Biosciences, NJ, USA), following the manufacturer’s instructions, and labelled with anti-Ki67 antibody (BD Horizon™ BV711 Mouse Anti-Human Ki67; BD Biosciences, NJ, USA) for 30 min at 4 °C in the dark. After washing twice with staining buffer, cell pellets were resuspended in 200 μl GLF16 diluent [95% PBS, 2.5% Tween-20 (Sigma-Aldrich®), 2.5% DMSO (PAN-Biotech GmbH)] containing 2 μl of the GLF16 dye (200 μg/ml) and incubated for 10 min at room temperature in the dark under mild shaking. After two washing steps with GLF16 diluent, samples were acquired on a Cytek Northern Lights spectral flow cytometer (Cytek® Biosciences) for stable flow cytometer performance, daily SpectroFlo® QC Beads (Cytek® Biosciences) were run. Data analysis was performed with FlowJo™ v10 Software (BD Life Sciences). The gating strategy for analysis is presented in the supplemental material. During the aforementioned analysis unstained controls were analyzed for all samples.

##### Sorting of live senescent cells applying mGLF16

Isolated PBMCs from the melanoma patients (Rs and NRs) were acquired according to Ficoll protocol as mentioned above. The cells were treated with 0.0166 μg/ml (Flow Cytometry) mGLF16 for 3 h (37 °C, 5% CO_2_). Cells were collected, washed, resuspended in PBS/0,5% FBS and then they were sorted [24]. The isolation of GLF16+ and GLF16-PBMCsp for subsequent RNA-sequencing was performed with a BD FACSMelody cell sorter (BD Biosciences, NJ, USA) using the high- purity mode. A minimum of 100.000 GLF16+ from NRs patients and GLF16- PBMCs from Rs patients were collected in separate tubes containing sorting buffer. Each sorted population was tested for contamination by a post-sort acquisition, which verified 99% purity for each sample.

##### Senescence assessment in tissues

Senescence assessment in FFPE samples was carried out using GL13 or GLF16 senoprobes as described insections "GLF16/Immunofluoresence" and "Immunocytochemistry-Immunohistochemistry". 

### Quantification and statistical analysis

In each experiment, values are demonstrated as means ± standard deviation. Differences between groups were estimated using the parametric 2-tailed Student’s t test, Wilcoxon test, the non-parametric Mann Whitney or 1-way ANOVA with Bonferroni’s post hoc test for multiple comparisons, as appropriate. p < 0.05 were considered significant. In order to compare the ages of Rs and NRs melanoma patients, a Shapiro–Wilk normality test was conducted to examine the normality of the distributions of the age in each group (p < 0.05, not normally distributed) and then a Wilcoxon test was held between the ages of the two groups of patients. Statistical analysis was performed using the Statistical Package for Social Sciences (SPSS) version 13.0.0 (International business machines-IBM).

## Results

### Decoding the senescence molecular signature SeneVick

We have recently generated SeneVick by incorporating studies that assessed cellular senescence in human cells using the senescence detecting algorithm (SDA) and concurrently included a variety of high throughput data (transcriptomics: RNA-seq and scRNA-seq, proteomics and epigenomics [14], Figure S1a). The signature is composed of 100 genes and exhibits an expression motif that complies with the senescence phenotype. The majority of them are down-regulated (n = 67) (Figure S1b). Characteristically, the genes included in the signature are implicated in diverse biological processes and can be grouped into distinct functional clusters, reflecting to a large extent the hallmarks of the senescence phenotype [1] (Figure S1b). The most profound cluster regards genes encoding potent cell cycle regulators and factors involved in the cellular response to DNA damage (Figure S1b). The second one consists of genes controlling chromosome structure and stability (Figure S1b). A considerable proportion of the signature encompasses genes related to the activation of immune responses and interactions among immune cells, while the other two clusters contain genes involved in metabolic and other functions (Figure S1b). Further zooming into clusters uncovered the involvement of the genes in a variety of cellular processes (Figure S1c). Interestingly, common genes between SeneVick and state of the art senescence molecular signatures, namely the SenMayo and FridMan gene sets, were identified [22, 23] (Figure S1d).

Our senescence signature exhibits several unique and biologically intriguing features that underline its potential functional importance. First and foremost, analysis of the SeneVick gene set revealed that the constituent genes do not represent simple haplotypes, suggesting that their co-occurrence is not the result of genetic linkage or population-based inheritance patterns. Another prominent molecular feature of the SeneVick-encoded proteins is their strong enrichment in ankyrin repeat domains. Ankyrin repeats are highly structured motifs that mediate protein–protein interactions, often serving as scaffolds in large signaling complexes [51]. Their consistent appearance across nearly all proteins encoded by the SeneVick genes suggests a non-random, biologically meaningful pattern, potentially orchestrating the senescence phenotype. Indeed, ankyrin repeat-containing proteins have been implicated in the regulation of cellular integrity, cell-cycle arrest, stress signal transduction, and differentiation processes, all of which have been linked with cellular senescence [51]. In addition to structural motifs, the genomic architecture of the SeneVick genes revealed another layer of functional organization: a statistically significant enrichment in T-dimeric motifs within their genomic sequences, exhibiting a periodic distribution. Given that periodic nucleotide motifs have been associated with the dynamic modulation of gene expression, this feature raises the possibility of a regulatory role during senescence [52]. Lastly, when mapping the SeneVick genes on chromosomes, we identified their absence in chromosomes 14, 18 and 21 and their underrepresentation in chromosome 13 (Figure S1b). Interestingly, these chromosomes are associated with trisomy syndromes—Patau (trisomy 13), Edwards (trisomy 18), and Down syndrome (trisomy 21) or lethality (trisomy 14), entities characterized by premature aging, chronic inflammation and senescence phenotypes [53–55]. It could be hypothesized that the absence or underrepresentation of SeneVick genes at these chromosomes may imply a protective genomic architecture, where senescence regulators are compartmentalized away from the chromosomal loci whose abnormal dosage leads to accelerated aging syndromes or premature death.

### SeneVick effectively identifies senescence irrespective of tissue origin, senescence type or species

**Fig. 1:**
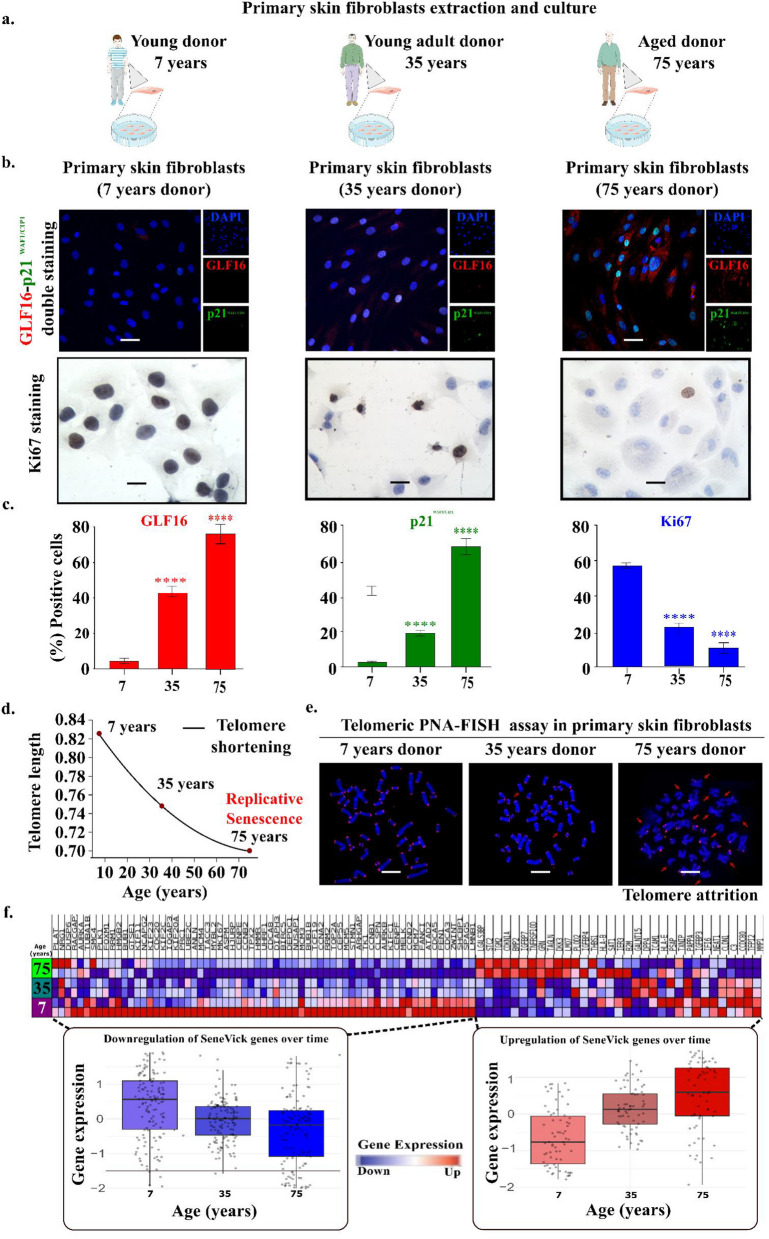
Validation of SeneVick in a human replicative senescence (aging) model. **a.** Schematic illustration of the primary skin fibroblasts extraction and culture. **b** Upper panel: Senescence assessment in human primary fibroblasts from different age groups (Age: 7-, 35-, and 75 years) using the senescence detecting algorithm (SDA). Representative images of double staining of cells with the senescence markers GLF16 (red) and p21^WAF1/CIP1^ (green) and DAPI counterstain. The images were quantified using ImageJ (*n* = 3 biological replicates). Objective: 20x. Scale bar: 30 μm. Lower panel: Evaluation of proliferation in human primary fibroblasts from different age groups (Age: 7-, 35-. and 75-years). Representative images of Ki67 immunocytochemical staining (upper panel). Positive cells were calculated by evaluating the strong brown nuclear signal for Ki67. **c.** Graphs depict the percentage of positive cells (%) for GLF16 (right side), p21.^WAF1/CIP1^ (middle side) and Ki67 (right side). Approximately 100 cells per optical field were counted, and ≥ 5 high-power fields per sample were used for the quantification. Statistical analysis was performed employing Wilcoxon nonparametric test. The data obtained represent means ± standard deviation. *P* < 0.05, **P < 0.01, ***P < 0.001, ****P < 0.0001. Objective 20×, 40×. Scale bars: 30 μm and 60 μm respectively. **d.** Telomere length curve depicting telomere attrition during aging in human primary fibroblasts. **e**. Microscopy images from PNA-FISH using DAPI with a telomere-specific probe (right) depicting telomere attrition on metaphase chromosomes from the three age groups. Objectives 63x. **f.** Human fibroblast gene expression after VST-transformation and z-scaling, (left panel = upregulated genes, right panel = downregulated genes). The upregulated genes show progressive increase, whereas the downregulated genes show progressive decline. This result is in concordance with the result of the GSEA analysis. The corresponding heatmap depicts duplicates for each age group. Two data sets were compared with unpaired t-tests, *P < 0.05, **P < 0.01, ***P < 0.001, ****P < 0.0001. "Donor icons were provided by Servier Medical Art (https://smart.servier.com/), licensed under CC BY 4.0 (https://creativecommons.org/licenses/by/4.0/)"

The SeneVick signature emerged by exploiting data from human cells of different tissue origin and proved efficient in demarcating non-senescence from senescence and in discriminating cellular senescence from aging in the liver [14]. We subsequently focused on testing its applicability and validating its fidelity in detecting senescence across tissues in other species besides humans. To elucidate this, we initially applied SeneVick in a publicly available single-cell transcriptomic atlas that was extracted across the lifespan of *Mus musculus* [19]. This dataset comprised scRNA-seq data obtained from 20 tissues and organs of mice split into six age groups, that ranged from 1 month which is equivalent of human early childhood to 30 months (equivalent to a human centenarian) [19]. Given that single cell analyses allow for the determination of gene expression in specific cell populations, they can facilitate uncovering certain cellular processes, such as cellular senescence, that might have been overlooked or hidden upon bulk RNA analyses. As demonstrated in Figure S2a, SeneVick was found significantly enriched in a variety of cell types and most profoundly in cardiac fibroblasts, keratinocytes and skeletal muscle cells of old mice (18 months and beyond, Wilcoxon, *p* < 0.05), reaching the highest values in older mice (Figure S2b). These results are in line with other studies in the same tissues supporting a linear increase of the proportion of cells expressing senescence markers with age progression [19]. The latter was further confirmed in an *in vitro * human setting consisting of primary skin fibroblasts obtained from 7-, 35-, and 75-year-old individuals, capturing time-points of the aging process. In contrast to young cells, those from 75 year old donors were found to exert replicative senescence (a senescence type induced by telomere shortening) [1] that was verified applying the SDA along with telomere length analyses (Fig. 1a, 1b,1c, 1d, 1e). To crosscheck this finding, we isolated RNA from these cells and implemented SeneVick in RNAseq data extracted from these fibroblasts and identified a progressive enrichment of the signature upon replicative senescence and age (Fig. 1f). Particularly, GSEA analysis resulted in a positive Normalized Enrichment Score (NES) of 1.76 (*p* < 0.002) for genes whose expression increases with age and a negative NES of −3.18 (*p* < 0.001) for those that are downregulated as age progresses (Fig. 1f). Overall, these findings highlight the potency of the extracted signature in detecting senescence, irrespective of tissue origin and species.

**Fig. 2:**
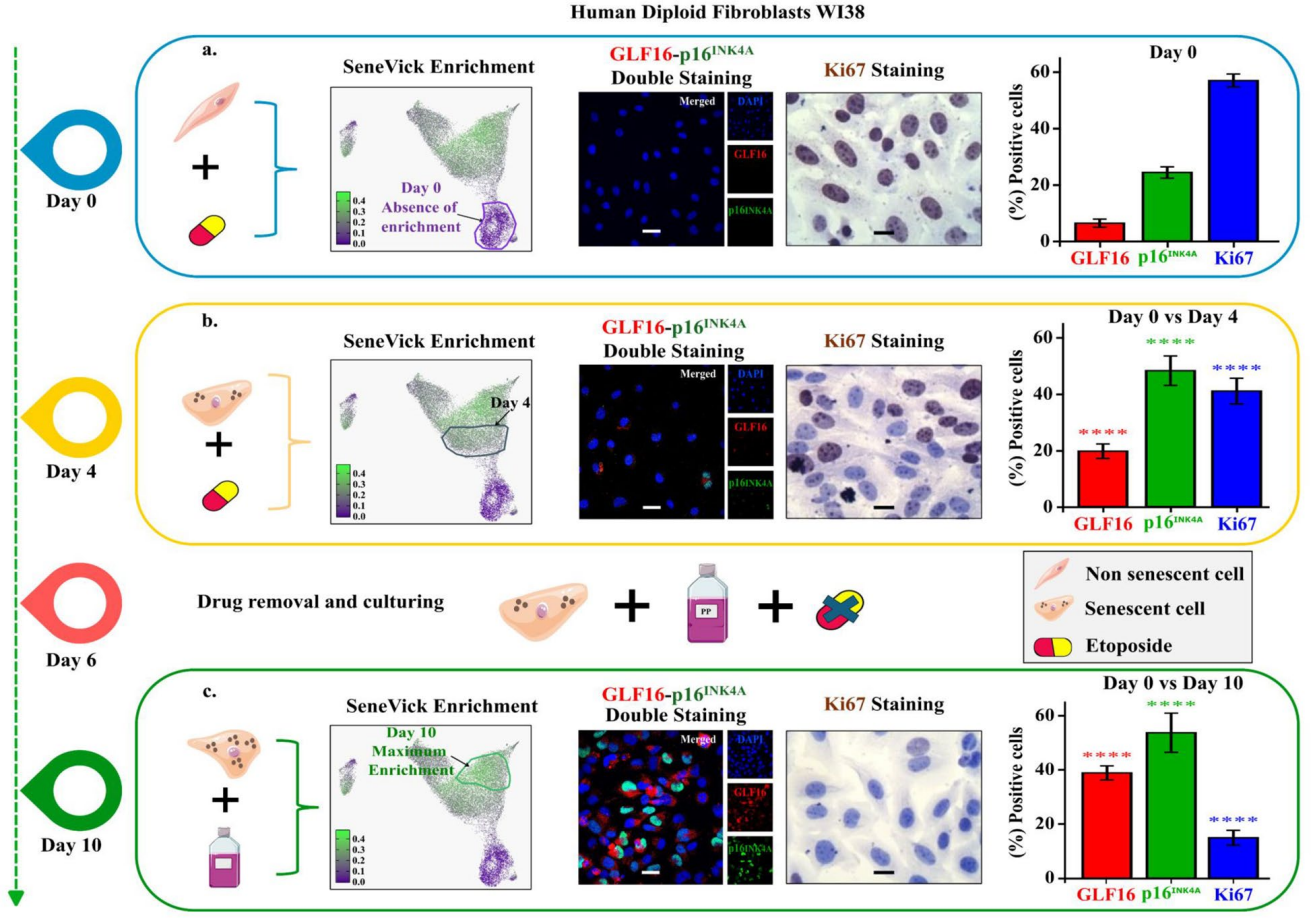
Validation of SeneVick in a human therapy-induced senescence model. **a** Left panel: Schema depicting the experimental procedure followed in day 0 to establish etoposide-induced cellular senescence in human fibroblasts. Middle panel: UMAP plot of human fibroblast scRNA data (GSE226225) displaying their clustering upon time (days), following etoposide treatment. Right panel: Senescence assessment in human WI-38 fibroblasts in day 0 using the senescence detecting algorithm (SDA). Representative images of double staining of cells with the senescence markers GLF16 (red) and p16^INK4A^ (green) and DAPI counterstain. The images were quantified using ImageJ (*n* = 3 biological replicates). Objective: 20x. Scale bar: 30 μm. Images of Ki67 immunocytochemical staining (middle side). Positive cells were calculated by evaluating the strong brown nuclear signal for Ki67. Graphs (right side) depict the percentage of positive cells (%) for GLF16, p16^INK4A^ and Ki67. Approximately 100 cells per optical field were counted, and ≥ 5 high-power fields per sample were used for the quantification. Statistical analysis was performed employing Wilcoxon nonparametric test. The data obtained represent means ± standard deviation. *P* < 0.05, **P < 0.01, ***P < 0.001, ****P < 0.0001. Objective 20×, 40x. Scale bars: 30 μm and 60 μm respectively. **b.** Left panel: Schema depicting the experimental procedure followed in day 4 to establish etoposide-induced cellular senescence in human fibroblasts. Middle panel: UMAP plot of human fibroblast scRNA data (GSE226225) displaying their clustering upon time (days), following etoposide treatment. Right panel: Senescence assessment in human WI-38 fibroblasts in day 4 using the senescence detecting algorithm (SDA). Representative images of double staining of cells with the senescence markers GLF16 (red) and p16^INK4A^ (green) and DAPI counterstain. Images of Ki67 immunocytochemical staining (middle side). Graphs (right side) depict the percentage of positive cells (%) for GLF16, p16^INK4A^ and Ki67. The quantification of the images, the evaluation of the proliferation and the statistical analysis were accomplished as mentioned above (**a**). Lower panel: Schema depicting the culturing conditions to establish etoposide-induced cellular senescence in human fibroblasts across the 10 days of the experiment. **c.** Left panel: Schema depicting the experimental procedure followed in day 10 to establish etoposide-induced cellular senescence in human fibroblasts. Middle panel: UMAP plot of human fibroblast scRNA data (GSE226225) displaying their clustering upon time (days), following etoposide treatment. Right panel: Senescence assessment in human WI-38 fibroblasts in day 10 using the senescence detecting algorithm (SDA). Representative images of double staining of cells with the senescence markers GLF16 (red) and p16^INK4A^ (green) and DAPI counterstain. Images of Ki67 immunocytochemical staining (middle side). Graphs (right side) depict the percentage of positive cells (%) for GLF16, p16.^INK4A^ and Ki67. The quantification of the images, the evaluation of the proliferation and the statistical analysis were accomplished as mentioned above (**a**). "Fibroblast cells icons were provided by Servier Medical Art (https://smart.servier.com/), licensed under CC BY 4.0 (https://creativecommons.org/licenses/by/4.0/)"

To further validate SeneVick, we compared it with two state of the art senescence signatures, namely SenMayo and Fridman [22, 23]. Both signatures have been extracted from human data with the SenMayo gene set consisting predominantly of SASP factors, while the key genes represented in the Fridman signature are involved in six particular pathways: pRB/p53, cytoskeletal formation, interferon-related, insulin like growth factor-related, mitogen-activated protein kinase (MAPK) and oxidative stress. We implemented these three senescence signatures in a publicly available human sc-RNAseq dataset [20] (GSE226225) obtained from human fibroblasts that were analyzed in two different settings. First, sc-RNAseq data were extracted from a time course experiment by monitoring cells for 10 days following treatment with the chemotherapeutic drug Etoposide (ETO) (Fig. 2a). We repeated this experiment staining cells according to the SDA in three different timepoints [3] (Day 0, 4, 10). Both approaches revealed absence of SeneVick enrichment and lack of senescence markers in day 0 and a progressive increase in the following days, reaching the highest values at day 10 (Fig. 2b, Fig. 2c, Figure S3a). The *in silico* dataset was used to compare SeneVick with the other two signatures, taking into account the distribution of the enrichment scores [48] and using segmented linear regression analysis that allows the capturing of two different rates of increase. While the breakpoint was identified on or near day 1 in all signatures, the slope from day 0 to day 1 when applying SeneVick was higher (0.14) than the respective SenMayo (0.013) and Fridman (0.06) ones (*p* < 0.05, Figure S3b). Given that SeneVick was not found enriched in non-senescent (control) cells (day 0), this finding implies a larger difference of SeneVick enrichment between non-senescence and senescence (day 1 and beyond, Figure S3b). Similar observations emerged from the second setting where the signatures were applied in sc-RNAseq data of fibroblasts exerting different types of cellular senescence (irradiation-induced and ETO-induced). As shown in Figure S3c, enrichment of the SenMayo and Fridman signatures was also evident in the control (non-senescent) state while SeneVick was absent. Altogether, SeneVick is more specific and efficient compared to the other signatures in demarcating senescent cells from non-senescent ones, even when senescence is low providing thus a valuable tool to uncover senescence that might be encrypted or overlooked.

### Immune cell senescence drives response to immunotherapy in melanoma patients

Next, we tested whether immune cell senescence contributes to dysfunction of the immune cell compartment within the TME, affecting the outcome of immunotherapy. Particularly, we conducted a case control study, implementing two senescence detecting approaches that complement each other. First, SeneVick was retrospectively applied in a scRNA-seq dataset from melanoma patients that received immunotherapy [43, 44] and secondly, we performed the SDA in clinical material from an analogous melanoma cohort (Tables S2 and S3). We focused on melanoma based on the fact that immunotherapy is a first-line therapy in this type of malignancy, while in other types of cancer it is usually implemented in combination with chemotherapy or radiotherapy.

**Fig. 3:**
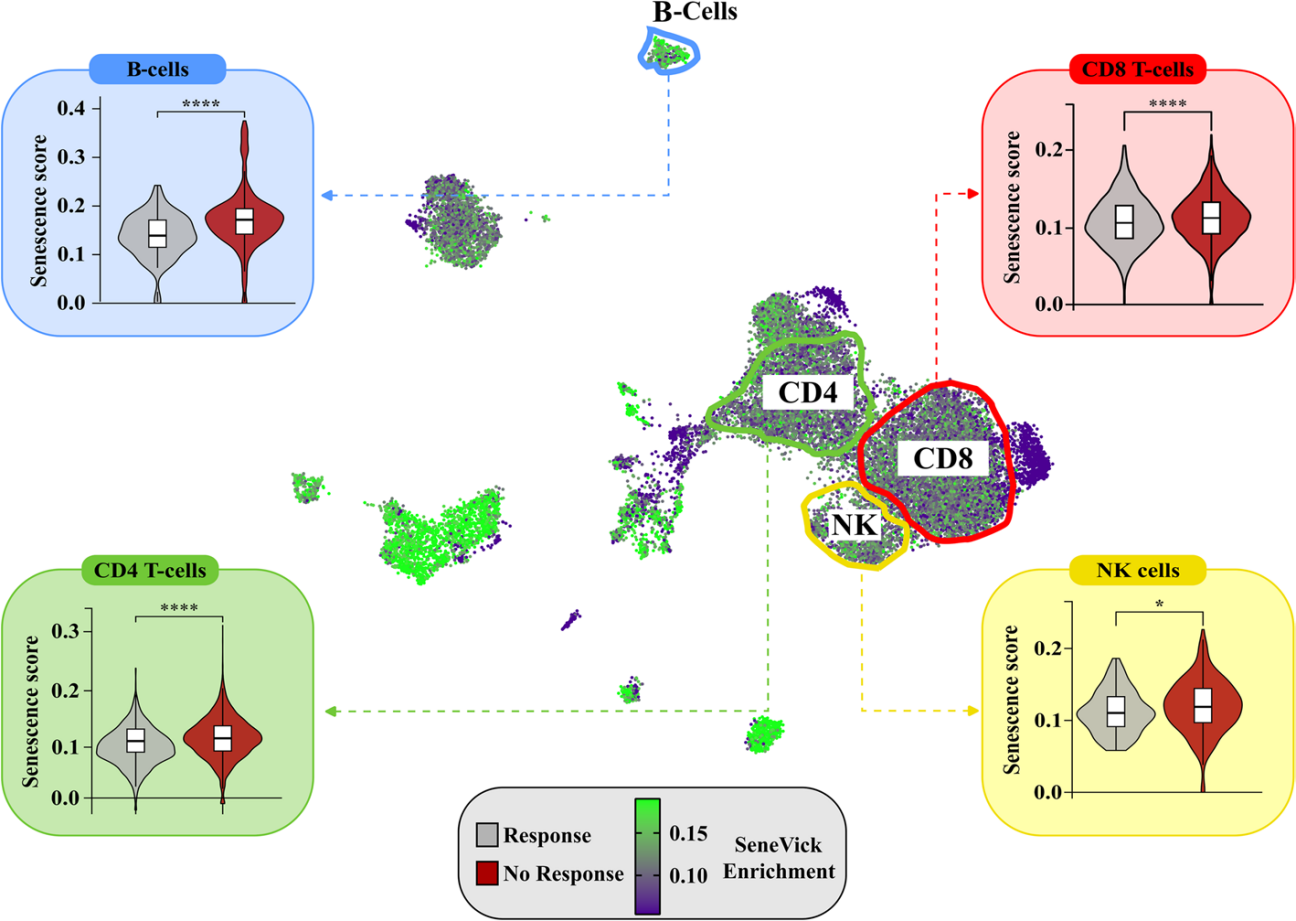
SeneVick implementation in an in silico melanoma dataset reveals increased senescent immune cell populations in NRs compared to Rs to immunotherapy. UMAP plot displaying the distribution of the cells from the single-cell RNA sequencing data of the 48 Rs and NRs melanoma patients following immunotherapy, from the GSE115978 (*n* = 30) and GSE120575 (*n* = 18) datasets. The cells are categorized based on SeneVick enrichment (middle panel). Violin plots show significant SeneVick enrichment in CD4 + and CD8 + T-cells, NK and B-cells (CD19 +/CD20 +) of NRs versus Rs (peripheral panels). To visualize the senescence scores, the scores of the cells below the 10th percentile and the ones above the 90th percentile were clipped, in order to reduce the influence of the outliers on the color scale. Nominal *p*-values were calculated via Wilcoxon test: CD8+ T-cells (*P* < 6.4e-08), CD4 + T-cells (*P* < 3.4e-06), B-cells (CD19 +/CD20 +) (*P* < 3.6e-08) and NK cells (*P* < 0.016). *P < 0.05, **P < 0.01, ***P < 0.001, ****P < 0.0001

**Fig. 4:**
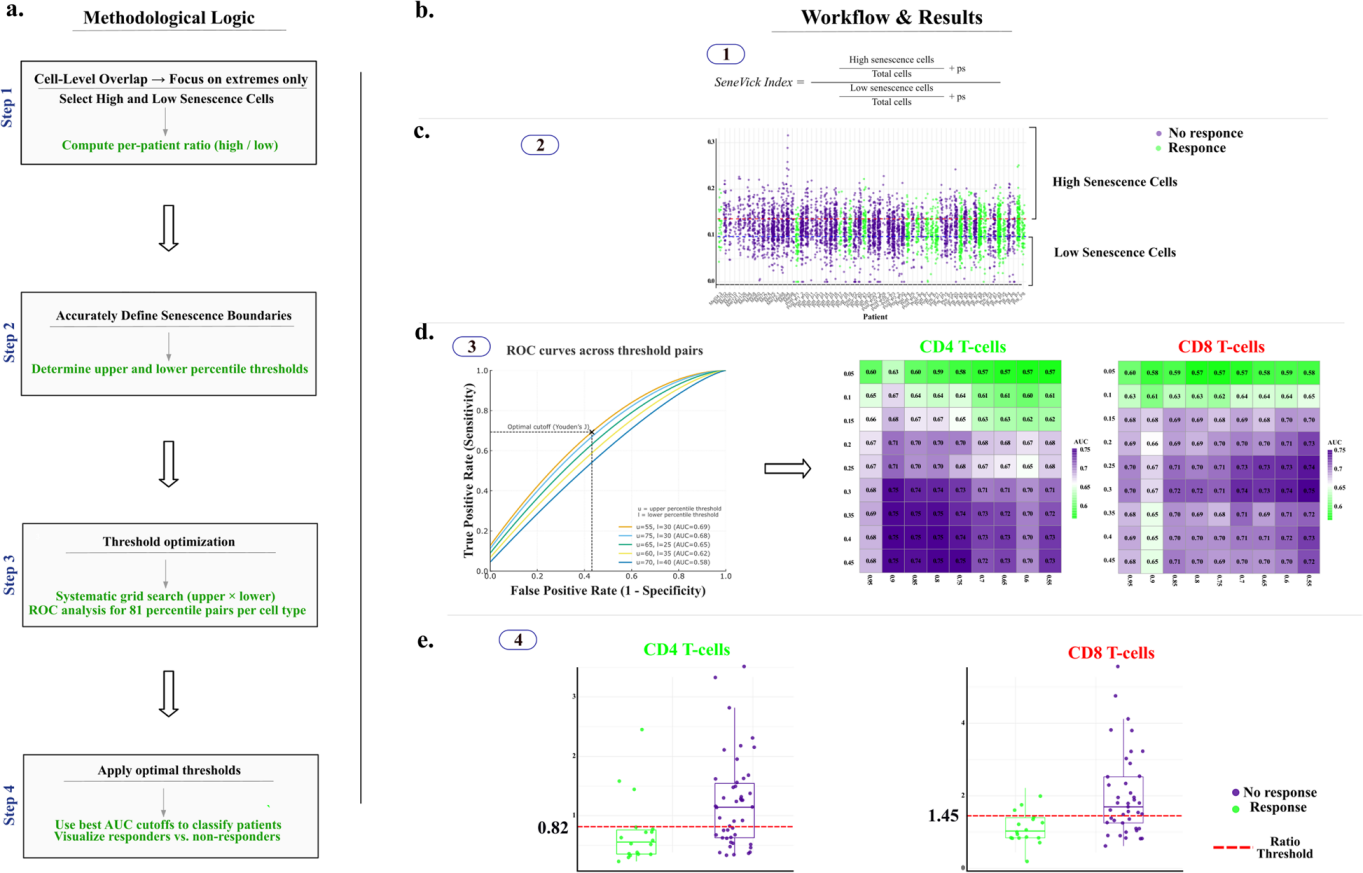
Workflow for applying SeneVick’s thresholding strategy to predict immunotherapy outcomes in Rs and NRs melanoma patients. **a** Methodological Logic (Steps 1–4). Schematic illustration of the pipeline used to achieve optimal senescence thresholds for our signature. **Step 1:** Reasoning/Logic behind the need for computing a per-patient ratio. **Step 2:** Senescence needs to be effectively defined, and its boundaries identified and set (quartiles method—upper and lower percentile thresholds for each cell type). **Step 3:** Perform systematic grid search and ROC analysis across all threshold pairs to identify the optimal upper-lower combination. **Step 4:** Apply the optimal thresholds to classify patients and visualize responders versus non-responders. **b.** The equation for calculating the SeneVick Index. **c.** Distribution of per-patient SeneVick enrichment values, dotted lines demonstrate the upper and lower percentile thresholds. **d** Schematic representation of the ROC-based threshold optimization workflow and the corresponding AUC scores generated heatmaps for CD4+ and CD8+ T-cells. **e.** Application of the optimal thresholds showing patient-level high senescent/low senescent ratios for CD4+ (left) and CD8+ (right) T-cells with the red dashed line denoting the cutoff values (Youden’s Index) separating Rs from NRs

Regarding the first approach we took advantage of the only two identified in the literature studies containing single cell data from melanoma patients following immunotherapy, particularly PD1, CTLA4 or combined inhibition and concurrently demonstrating the response status of these patients [43, 44] (Table S2). Thus, data regarding gene expression per cell type in Rs and NRs were accessible. In some cases, data obtained prior to treatment were also available (Table S2). As an initial step, we followed a detailed bioinformatic pipeline to integrate the two datasets as depicted in Figure S4, which included several steps of data processing, cell annotation and normalization. This process resulted in a cohort of a total of 48 melanoma patients comprising 30 NRs and 18 Rs. In this setting, we found distinct clusters of the immune cell compartment comprising mainly CD4 + T-cells, CD8 + T-cells, NK and B-cells (CD19 +/CD20 +). During the ensuing stages, we implemented SeneVick in the latter dataset and investigated the senescence status in each immune cell population and in relation to the response outcome. As demonstrated in Fig. 3, SeneVick enrichment was evident in CD4 + T-cells, CD8 + T-cells, NK and B-cells (CD19 +/CD20 +) and in relation to the response status we found that cells belonging to NR patients exhibited higher enrichment scores compared to responding patients. This phenomenon was independent of patients' age (p = 0.12) and other confounding factors such as age, sex, melanoma type and stage (Table S3). Next, we questioned whether we could assess an enrichment threshold value for SeneVick that can discriminate Rs from NRs and predict treatment outcome in our dataset. As explained in section "SeneVick's cut-off determination to predict immunotherapy response", we tested 81 upper and lower threshold combinations per cell type, in order to find the threshold pair which allows for the optimal discrimination of Rs and NRs. Specifically, in CD8 + T-cells, an upper threshold of 55 and a lower threshold of 30 yielded an AUC = 0.75 with an optimal index cutoff of 1.45. In CD4 + T-cells, an upper threshold of 75 and a lower threshold of 30 gave an AUC = 0.73 with an index cutoff of 0.82. NK and B-cells (CD19+/CD20+) were excluded due to low cell numbers (less than 10) and excessive patient loss. SeneVick's observed predictive performance reflects the contribution of senescence to immunotherapy resistance, in line with its established role as a key determinant of treatment outcome. Collectively, these findings substantiate the predictive value of the SeneVick signature in melanoma immunotherapy response (Fig. 4).

**Fig. 5:**
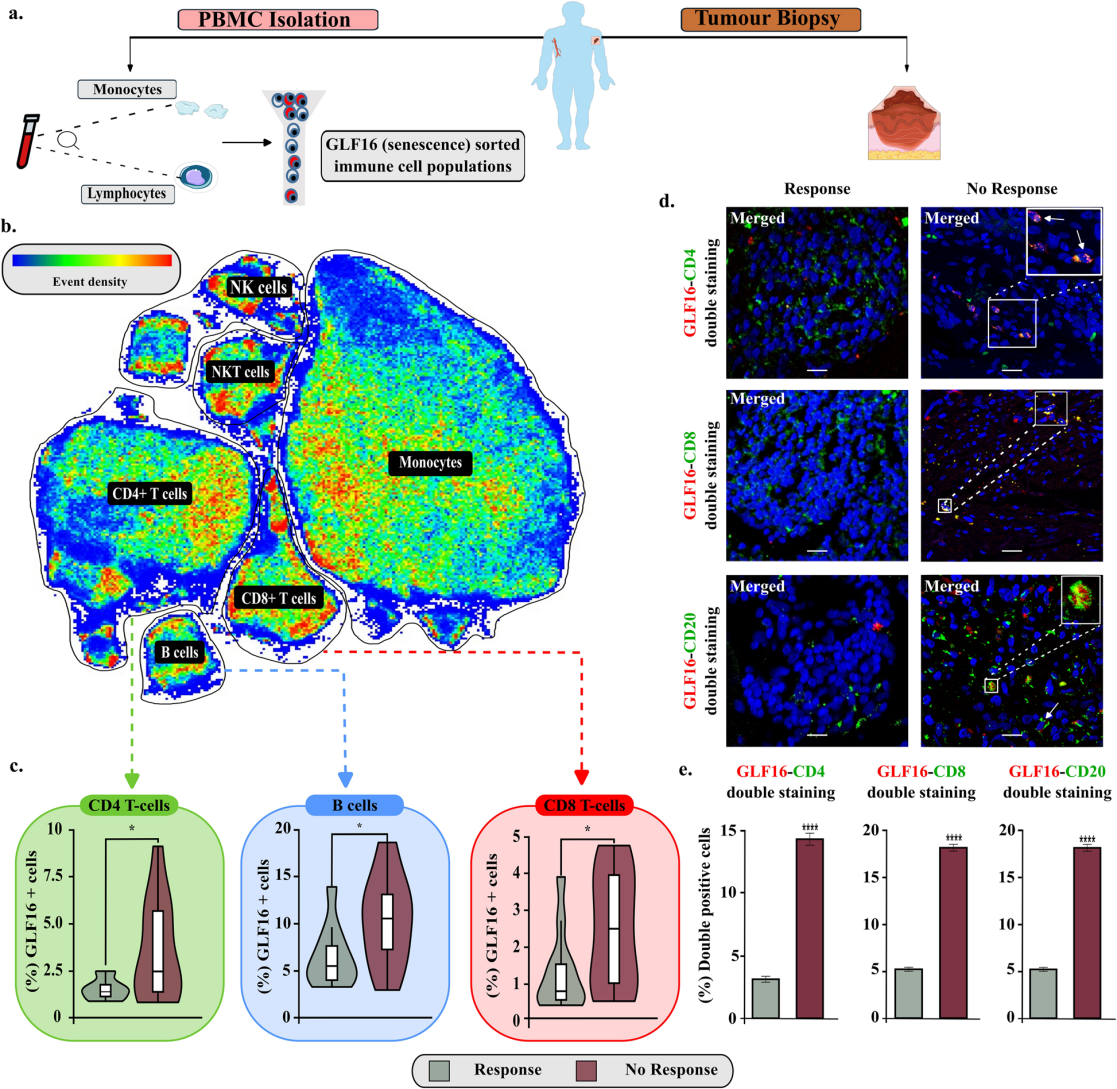
Senescence assessment in an *ex vivo* melanoma cohort, consisting of peripheral blood and tissues, demonstrates increased immune cell senescence in NRs compared to Rs to immunotherapy. **a.** Schematic overview of the experimental procedure followed to collect peripheral blood samples and tumor lesions from melanoma patients. Blood samples were processed with Ficoll-Paque density gradient centrifugation to isolate PBMCs. The latter were stained with a panel of fluorochrome-conjugated antibodies (Table S4) GLF16 and further analyzed with flow cytometry to assess GLF16+ (senescent) immune cell populations. In turn, tissue samples were double stained with GLF16 and immune cell markers to assess immune cell senescence. **b**. Representative UMAP plot showing the clusters of the major PBMC subsets, i.e., CD4+ T cells, CD8+ T cells, B-cells (CD19+/CD20+), NK cells, NKT cells, and monocytes. Color coding reflects event density (blue, low; red, high). **c.** Violin plots display the percentages of GLF16+ (senescent) CD4 + and CD8 + T-cells and B-cells (CD19+/CD20+), of responders and non-responders, *P < 0.05. **d** Representative images of double staining of cells with the senescence marker GLF16 (red) and CD4 (green), CD8 (green) and CD20 (green). DAPI counterstain. Scale bar: 30 μm. The images were quantified using ImageJ. **e.** Quantification of images presented in d. Statistical analysis was performed employing unpaired t-test. The data obtained represent means ± standard deviation. *P* < 0.05, **P < 0.01, ***P < 0.001, ****P < 0.0001. Objective 20×, Scale bar: 30 μm. "Melanoma biopsy, human and vessel icons were provided by Servier Medical Art (https://smart.servier.com/), licensed under CC BY 4.0 (https://creativecommons.org/licenses/by/4.0/":

**Fig. 6:**
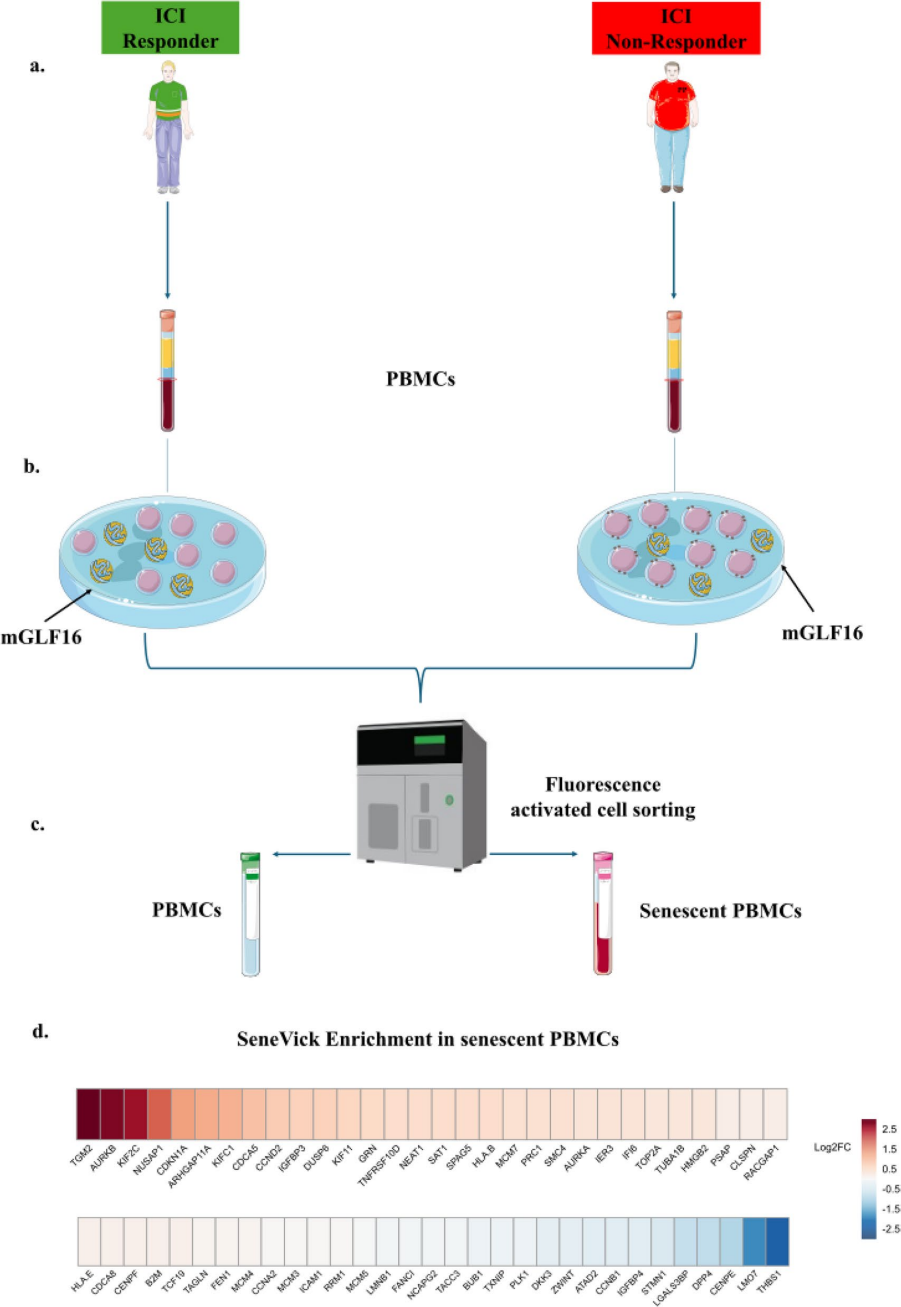
SeneVick’s utility in identifying immune cell senescence that drives immunotherapy outcome in Rs and NRs melanoma patients. **a. **Schematic overview of the experimental procedure followed to collect peripheral blood samples from Rs and NRs melanoma patients. These blood samples were processed with Ficoll-Paque density gradient centrifugation to isolate PBMCs. **b.** The PBMCs of Rs and NRs melanoma patients were divided into two groups that were cultured and stained with mGLF16 according to the SDA. **c.** RNA extraction from sorted GLF16 + (senescent) PBMCs of our NR melanoma patients.** d.** SeneVick implementation in these RNA datasets showing a clear enrichment of the signature and additionally signifying the senescent nature of these immune cells in NRs

The second approach included analysis of *ex vivo* clinical material from melanoma patients that received single or combined immunotherapy, as monotherapy (Table S3). Particularly, PBMCs from Rs and NRs patients were obtained and senescence was assessed via flow cytometry, using the GLF16 senoprobe, an essential component of the SDA [3–5]. This approach was favored as circulating immune cells have been demonstrated to reflect to a large extent the tumor infiltrating ones and provide a reliable snapshot of the TME [56]. As expected, PBMCs originating from NR patients exhibited significantly higher senescence particularly in the CD4+ and CD8+ T-, and B-cell (CD19+/CD20+) subtypes compared to Rs (Fig. 5, Figures S5-S6). This was also the case for NK cells, though the lack of statistical significance was probably due to the low number of *ex vivo* samples. Increased senescence in CD4+ and CD8+ T-, and B-cells (CD19+/CD20+) of NRs was subsequently confirmed in corresponding tissue biopsies from these patients using the SDA, additionally suggesting that NRs exert considerably increased immune cell senescence, in relation to Rs (Fig. 5, Figure S7). No association of immune senescence in NRs with clinical features presented in Table S3 was identified. Overall, the observations from the *in silico * and experimental analysis robustly support that NRs to immunotherapy can be distinguished from Rs based on their immune cell senescence status, irrespective of their age. Subsequently, RNA isolated from sorted GLF16+ (senescent) PBMCs of NR melanoma samples was enriched for SeneVick further strengthening the above findings (Fig. 6).

**Fig. 7:**
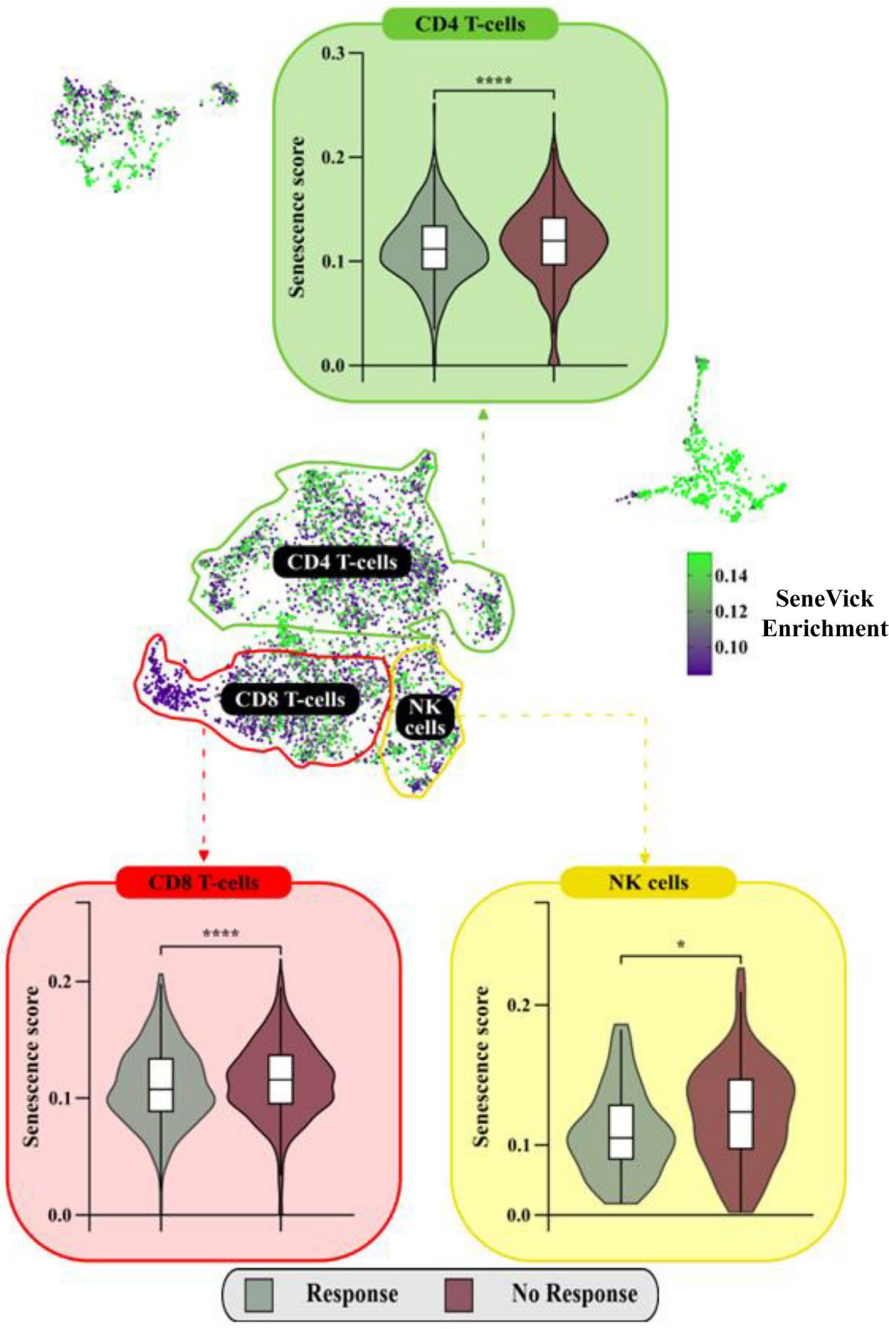
SeneVick identified significantly increased senescence in immune cell populations in NRs vs Rs prior treatment. UMAP plot displaying the distribution of the cells from the single-cell RNA sequencing data of the Rs and NRs melanoma patients prior to immunotherapy treatment, from the GSE115978 and GSE120575 datasets. The cells are categorized based on SeneVick enrichment (middle panel). Violin plots show significant SeneVick enrichment in CD4+ and CD8+ T-cells, and NK cells of NRs versus Rs (peripheral panels). To visualize the senescence scores, the scores of the cells below the 20th percentile and the ones above the 80th percentile were clipped, in order to reduce the influence of the outliers on the color scale. Nominal *p*-values were calculated via Wilcoxon test.: CD8+ T-cells (*P* = 0.00065), CD4+ T-cells (*P* = 6.1e-5) and NK cells (*P* = 0.011). *P < 0.05, **P < 0.01, ***P < 0.001, ****P < 0.0001

To exclude the possibility that immune checkpoint inhibition could be responsible for senescence observed in the immune cell populations, we implemented SeneVick exclusively in single cell data from melanomas before their treatment. We confirmed that NRs exerted a significantly increased senescence score in CD4+ T-cells (*P* = 6.1*10^–5^), CD8+ T-cells (*P* = 6.5*10^–5^) and NK cells (*P* = 0.011) compared to Rs, prior immunotherapy (Fig. 7). In B-cells (CD19+/CD20+) the difference was not statistically significant, most probably due to the small number of cells of these populations when only the pre-treatment samples are considered.

**Fig. 8:**
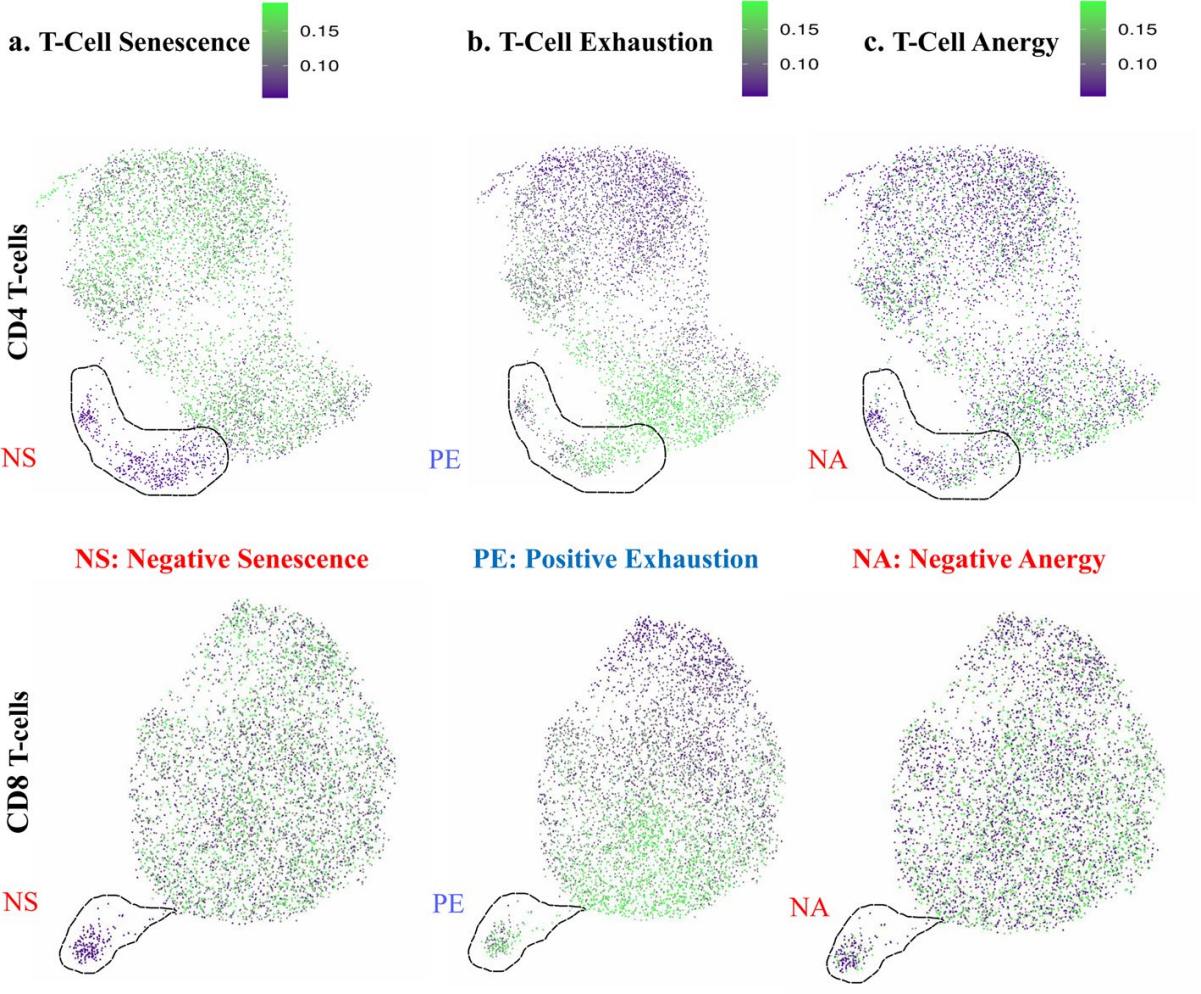
SeneVick effectively discriminates Senescence from Exhaustion and Anergy in the melanoma TME. UMAP plot displaying the distribution of the cells from the single-cell RNA sequencing data of the CD4+ and CD8+ T-cells of the 48 Rs and NRs melanoma patients and categorizing cells after (**a**) the implementation of SeneVick (**b**) the T-Cell exhaustion signature and (**c**) the T-Cell anergy signature. As outlined, a subset of non-proliferating CD4+ and CD8+ T-cells in NRs exerts absence of enrichment of SeneVick and the T-cell anergy signature while exhibiting a T-cell exhaustion phenotype. To visualize the signature scores, the scores of the cells below the 10th percentile and the ones above the 90th percentile were clipped, in order to reduce the influence of the outliers on the color scale

Interestingly, within the CD4+ and CD8+ T-cell subsets of NRs, a relatively small population of cells that was neither enriched for SeneVick nor proliferating drew our attention. Further zooming into this observation, we questioned whether these cells could be exhausted or anergic, as these T-cell states have been previously reported as a source of immune cell dysfunction [57]. In order to examine this issue, we extracted two signatures consisting of the most potent markers identified in the context of exhaustion and anergy respectively (Table S5) and applied them in the CD4+ and CD8+ T-cell compartment of our melanoma cohort. Indeed, cell populations negative for senescence, anergy and proliferation exerted an increased enrichment of the "exhaustion" signature while those showing SeneVick enrichment were simultaneously negative for exhaustion, anergy and proliferation (Fig. 8). These observations highlight the fidelity of SeneVick in distinguishing cellular senescence from other dysfunctional cell states within the immune cell compartment, thus allowing the elucidation of its role not only in cancer but also in a variety of other diseases.

**Fig. 9:**
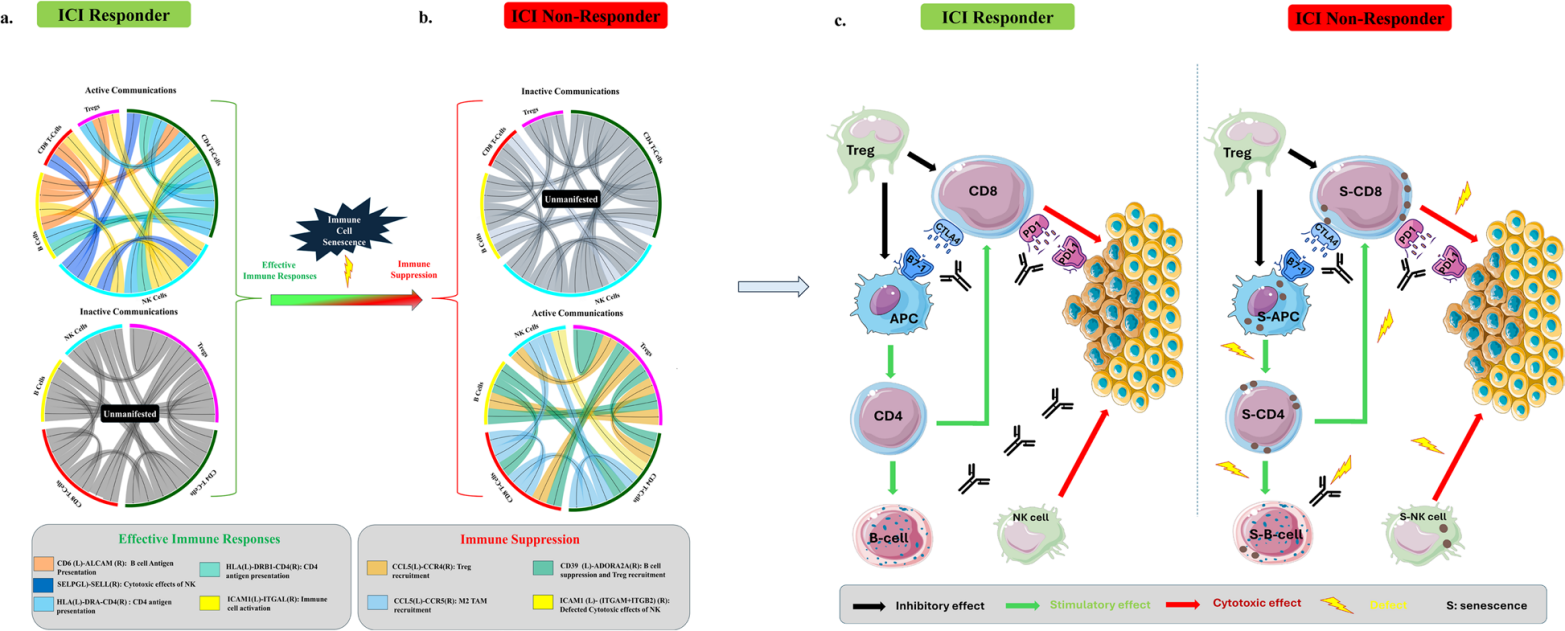
Analysis of the intercellular interplay of the immune cell subpopulations in Rs versus NRs melanoma patients following immunotherapy. **a**. Cell chat derived circular representation of cell-to-cell interactions (CD4+ T-cells, CD8+ T-cells B-cells, NK cells and Tregs) which are active in the ICI responder group and are responsible for the effective immune responses depicted. Each arrow displays the Ligand-Receptor (L-R) interactions for the different ligand-sender cell types. The main events promoting effective immune responses in Rs include: active antigen presentation [58, 59], NK cells penetration through the vasculature enhancing their cytotoxic effects [60] and increased cytoxicity through activation of various immune cell populations [61]. **b.** Cell chat derived circular representation of cell-to-cell interactions (CD4+ T-cells, CD8+ T-cells, B-cells, NK cells and Tregs) which are active in the ICI Non-Responder group and are responsible for the ineffective immune responses depicted. Each arrow displays the L-R interactions for the different ligand-sender cell types. Among the mechanisms involved in immunodeficiency in NRs are: increased cAMP production that leads to B-cell suppression and Treg recruitment [62], increased Treg accumulation resulting in blockage of cytotoxic responses [63], increased attraction of M2 tumor-associated macrophages inducing T-cell inhibition [64, 65] and impairment of NK circulation and extravasation driving defective NK cytotoxic effects [66]. **c.** Schematic illustration representing the altered immune cell interactions in NRs compared to Rs following immunotherapy. "Cell icons were provided by Servier Medical Art (https://smart.servier.com/), licensed under CC BY 4.0 (https://creativecommons.org/licenses/by/4.0/)"

**Fig. 10:**
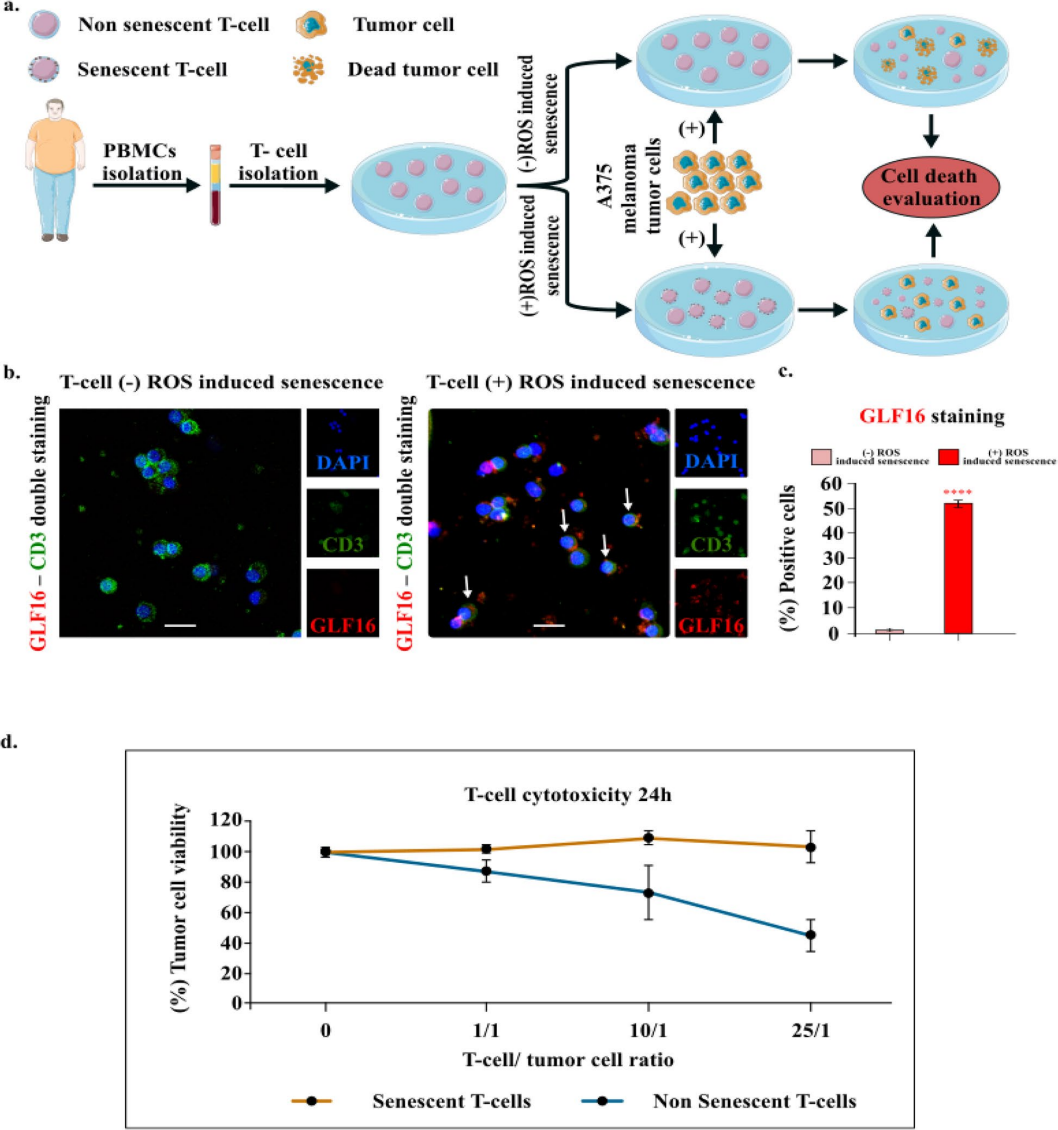
Ineffective immune responses of senescent T cells against melanoma cells. **a** Schematic overview of the experimental procedure followed to collect peripheral blood samples from a young donor (left panel) and their co-culture with A375 melanoma cells. This blood sample was processed with Ficoll-Paque density gradient centrifugation to isolate PBMCs. In the following steps, the PBMCs were stratified into two subgroups, with the second one being subjected to oxidative stress-induced cellular senescence. The two subgroups were co-cultured with A375 melanoma tumor cells (right panel) in order to evaluate their cytotoxic abilities. **b** The two aforementioned groups of PBMCs were stained with GLF16 according to the SDA and CD3. Representative images of double staining of cells with the senescence marker GLF16 (red) and CD3 (green), DAPI counterstain. Scale bar: 30 μm. The images were quantified using ImageJ (n = 3 biological replicates). **c.** Graphs (right side) depict the percentage of positive cells (%) for GLF16. Approximately 100 cells per optical field were counted, and ≥ 5 high-power fields per sample were used for the quantification. Statistical analysis was performed employing Wilcoxon nonparametric test. The data obtained represent means ± standard deviation. *P* < 0.05, **P < 0.01, ***P < 0.001, ****P < 0.0001. **d** T-cell cytotoxicity assays from the corresponding co-cultures of ROS-induced senescent T-cells and untreated T-cells. "Cell icons and blood samples were provided by Servier Medical Art (https://smart.servier.com/), licensed under CC BY 4.0"

Lastly, in order to gain further insights into the alterations that senescence may impose in the TME of NRs, we analyzed the intercellular interplay of the immune cell subpopulations in Rs versus NRs. Particularly, in the formerly analyzed melanoma scRNA dataset of Rs and NRs patients we applied a cell communication analysis using CellChat [50]. The results showed numerous differences of ligand-receptor communication probabilities among the immune cell types previously encountered (CD8+ and CD4+ T-cells, NK cells, and B-cells (CD19+/CD20+) (Fig. 9a). Further exploiting these data, we identified cell-to-cell interactions that promote effective immune responses and are intact in Rs and dysfunctional in NRs as well as immune cell interplays that favor an immunosuppressive environment and are active in NRs and inactive in Rs (Fig. 9b**, **Figure S8). The main events promoting effective immune responses in Rs include: active antigen presentation [58, 59], NK cells penetration through the vasculature enhancing their cytotoxic effects [60] and increased cytotoxicity through activation of various immune cell populations [61]. On the other hand, among the mechanisms involved in immunodeficiency in NRs are: increased cyclic adenosine monophosphate (cAMP) production that leads to B-cell suppression and Treg recruitment [62], increased Treg accumulation resulting in blockage of cytotoxic responses [63], increased attraction of M2 tumor-associated macrophages inducing T-cell inhibition [64, 65] and impairment of NK circulation and extravasation driving defective NK cytotoxic effects [66] (Figure S8). Notably, deregulated expression of the ligand-receptor interactions presented in Figure S8 has been reported in the context of non-immune senescence cell types [1, 67–69]. In line with the above, we demonstrated that senescent T-cells display impaired cytotoxic activity in relation to their non-senescent counterparts (Fig. 10). To the best of our knowledge this is the first time shown that senescent T-cells exhibit dysfunctional properties and drive defective immune responses. All the above and the fact that among the immune cell dysfunctional states the population of senescent cells was the most prevalent one in the scRNA NR subset (Fig. 8), support immune cell senescence as an important determinant for responsiveness to immunotherapy.

## Discussion

Immunotherapy has undoubtedly provided major benefits towards cancer treatment in the last decades, though in a significant portion of patients the treatment yields limited or even no responses [17]. Cancer-related immunodeficiency has arisen as an important determinant of this outcome [70–72]. The latter has been linked to the accumulation of dysfunctional immune cell populations within the TME, such as exhausted and anergic ones while the contribution of cellular senescence is still poorly understood [73–76]. In the context of immunity, senescence has often been inaccurately assessed due to the application of debatable and non-specific markers [5]. As a consequence, the term immune cell senescence has been misused, its contribution to shaping the TME is still vague and probably underestimated, and its association with the clinical course remains largely unknown [5]. In line with this notion, given that the senescence phenotype is complex and highly heterogeneous, the development of new approaches to assess it more accurately complementing existing or evolving tools, will enhance our understanding on the cellular and molecular mechanisms involved, and its impact on human diseases and clinical outcomes [13, 77].

The current study investigated whether cellular senescence is a source of immune cell dysfunction and is involved in the response to immunotherapy by applying two approaches that complement each other, an in silico one as well as a guideline senescence detecting pipeline (SDA), in melanoma patients. Melanoma was selected, as this type of malignancy is commonly treated with immunotherapy as a first option, especially in advanced stages. Moreover, this type of treatment, as shown (Fig. 7) is not linked to therapy-induced senescence in the immune cell compartment, in contrast to what is observed in traditional ones (irradiation or chemotherapy) that cause DNA damage-induced senescence and thus did not influence our observations [78, 79]. Regarding the first approach, we initially extensively validated SeneVick, a senescence molecular signature comprising 100 genes that we recently extracted, allowing the discrimination of senescence from aging in the liver [14] (Figure S1). The signature turned out to be highly sensitive and specific in discriminating non-senescent cells from senescent ones, irrespective of senescence type, tissue origin or species, even upon low senescence levels (Figs. 1– 2, Figures S3-S4). Of note, SeneVick is not a stochastic gene set, but rather a highly structured and evolutionarily curated network of genes likely acting in coordination to mediate key aspects of cellular senescence (Figure S1). The combination of rare protein domains, motif periodicity, and chromosomal distribution patterns not only provides insights into the mechanistic underpinnings of senescence but also hints at the potential translational value of SeneVick in biomarker development, aging research, and therapeutic interventions targeting age-related pathologies.

We next exploited this specific and potent senescence detecting tool, as the first approach, to address the main query of our investigation related to the potential involvement of tumor-related immune cell senescence in the outcome of immunotherapy. By implementing SeneVick retrospectively in a scRNA dataset from 48 melanomas treated exclusively with immune checkpoint inhibitors (PD1, CTLA4 or combined inhibition), we observed a considerable signature enrichment in the immune cell populations of NRs, comprising CD4+ and CD8+ T-cells, NK and B-cells (CD19+/CD20+), that was independent of patient’s age and other clinical features (Fig. 3). Furthermore, a workflow to determine an optimal cutoff value for discriminating between responders and non-responders was designed and applied, highlighting the predictive value of SeneVick in melanoma immunotherapy response (Fig. 4).

Subsequently, as the second approach, we verified the presence of senescent immune populations in peripheral blood mononuclear cells (PBMCs) from 11 Rs and 13 NRs melanoma patients following the SDA via flow cytometry [3–5] (Table S4). Indeed, PBMCs originating from NR patients, particularly CD8+ and CD4+ T-cells, and B-cells (CD19+/CD20+) exhibited remarkably higher senescence compared to those from Rs (Fig. 5). This was also the case for NK cells, though the differences were not statistically significant, putatively due to the low number of *ex vivo* samples available. Interestingly, higher senescence levels were confirmed in these immune cell types within the corresponding tumour lesions of the NR patients compared to Rs (Fig. 5). These observations were also in line with those that emerged following SeneVick implementation in the in silico dataset. Of note, although senescent populations identified in the peripheral blood were quantitatively lower, they reflected qualitatively those in the TME in each case and overall, confirming the value of circulating immune cells as a reliable setting to assess cellular senescence in patients [80, 81] (Fig. 5, Figure S7). Additionally, when applying SeneVick in sorted senescent PBMCs from NR melanoma patients, a clear enrichment of the signature was observed (Fig. 6). At this point it should be mentioned that SenMayo and Fridman exhibit drawbacks that restrain their applicability for senescence related studies [15]. SenMayo is heavily composed of SASP-related transcripts, many of which participate in various other cellular processes such as inflammatory, stress, or immune activation responses, leading to cross-reactivity and false-positive enrichment. Importantly, senescence regulators such as CDKN2A and CDKN1A are absent, limiting its diagnostic depth. Conversely, the Fridman signature was derived from literature-based meta-analysis rather than high-throughput omics data, and many of the original reference studies relied on indirect or non-specific senescence markers, including oxidative stress genes and cell cycle regulators, whose interpretation is now considered insufficient to define true senescence [3, 22, 23]. As a result, when applied to non-senescent control datasets, both SenMayo and Fridman showed spurious enrichment in the absence of senescence, whereas SeneVick correctly remained uninduced (Figure S3). This phenomenon likely reflects inflated type I error and "artificial enrichment," where broadly inflammatory or stress-responsive genes overlap with unrelated biological noise. In contrast, SeneVick, developed directly from datasets validated with the guideline SDA and the GLF16 fluorophore, integrates multi-omics data (transcriptomic, proteomic, epigenomic) anchored on experimentally verified senescent states [3, 14, 24]. This design ensures alignment between in silico and *in vitro* senescence detection, yielding high specificity and biological interpretability, overall signifying the superiority of SeneVick for senescence related studies.

Our findings underscore the importance of immune cell senescence in driving responsiveness to immunotherapy in melanoma and demonstrate that the tools implemented herein in a complementary manner are highly efficient to identify those patients who are likely not to respond according to their senescence status. While indications in experimental models support that tumor cell senescence might influence the outcome of immunotherapy, our study unveils for the first time the role of immune cell senescence within the TME in responsiveness to such an intervention in melanoma [82]. Interestingly, the *in silico* analysis identified a subset of non-proliferating CD4+ and CD8+ T-cells among NRs where SeneVick signature was not enriched (Fig. 8). These cells were found to exhibit a T-cell exhaustion signature and were devoid of anergy markers. In line with this notion, NR patients of the clinical melanoma cohort with the lowest senescence (GLF16) indices exerted characteristically the highest exhaustion levels. These findings denote the reliability of SeneVick and GLF16 staining in discerning cellular senescence from other dysfunctional T-cell states within the TME, a task that so far has been really challenging in phenotypic analyses due to significant overlapping of the applied markers [5, 24]. Moreover, numerous differences of intercellular communication among the immune cell populations (CD8+ and CD4+ T-cells, NK cells and Β-cells (CD19+/CD20+) between Rs and NRs, were identified (Fig. 9a, Figure S8). Overall, cell-to-cell interactions mediating effective immune responses were intact in Rs and dysfunctional in NRs while the interactions favoring an immunosuppressive context were found activated in NRs and inactive in Rs (Fig. 9b, Figure S8). Interestingly, their deregulation has been also identified in non-immune senescent cells [1, 68, 69]. In line with these findings, we also showed that senescent T-cells are incapable of eliminating tumor cells in relation to their non-senescent counterparts, associating directly T-cell senescence with impaired cytotoxicity (Fig. 10). Given that among the dysfunctional immune cell populations in the NRs the senescent one was the most prevalent (Fig. 8), underscores the role of senescence-mediated immune suppression in imposing resistance to immunotherapy (Fig. 9b). The latter is characterized by senescence in CD8+ T-cells, and NK cells leading to loss of their cytotoxic activity, while senescence in CD4+ T-cells that exert a multifaceted role in cancer immunity, leads to a diminished pool of functional T-cells incapable of responding to new antigens [83–85]. Senescent cell populations, particularly CD8+ and CD4+ T-cells, can secrete a variety of SASP factors (pro-inflammatory cytokines, chemokines and growth factors), that can remodel the immune landscape and influence the TME towards tumour progression [86–88]. In this context, tumour infiltrating senescent T lymphocytes can affect B-cell (CD19+/CD20+) activation and their subsequent differentiation into CD27+/CD38+ cells, ultimately leading to a deficiency in antibody production and inefficient adaptive responses [86–88]. The latter is also a potential outcome of B-cell (CD19+/CD20+) senescence as identified in our analysis in NRs. Regarding the limitations, our investigation should be regarded as a starting point aiming to unveil the role of immune cell senescence within the TME and its involvement in immunotherapy outcome. As such, it needs to be expanded not only in melanoma but also in a wide spectrum of other malignancies and irrespective of whether Immune Checkpoint Inhibition (ICI) is the first-line treatment or follows conventional ones that can trigger senescence [17, 89, 90].

While there seems to be a relation between aging and immune senescence [88], our findings suggest that cancer cells can shape a microenvironment to promote immune cell senescence as a strategy for immune evasion, independent of patients' age, addressing thus a debatable matter. Potential mechanisms involved are summarized in Figure S9. Nevertheless, these mechanisms should be regarded cautiously, as markers applied for senescence identification in these studies can also be evident in other immune cell dysfunctional states [5].

Given the plasticity of immune cells and that anergy or exhaustion reflect in principle progressive and irreversible states acquired upon chronic infections or cancer, immune cell senescence emerges as an attractive option to rejuvenate the immune system in order to restore its functionality, increasing thus the efficacy of immunotherapy. In fact, strategies for immune cell reinvigoration that target the above molecular mechanisms and pathways as well as the elimination of the toxic and immunosuppressive senescent cell compartment in the TME are gaining increased attention [5, 91]. Regarding the latter, a recently reported innovative senolytic platform that allows for selective removal of senescent cells without side effects paves the way [92]. This advancement underscores the importance of investigations such as the current one that exploits efficient senescence detecting tools to characterize patients according to their senescence status which drives their responsiveness to therapy.

## Conclusion

Immunotherapy has significantly improved cancer treatment. However, not all cancer patients benefit from such interventions, rendering the elucidation of differences between responders and non-responders at the molecular/cellular level an imperative task. Dysfunctional immune cell states such as T-cell exhaustion and anergy have been linked to failure of checkpoint inhibitors, while the role of immune cell senescence remains elusive. In the current study, we investigated this issue in melanomas where immunotherapy is applied as a first line treatment, following two senescence detecting complementary approaches. We found for the first time that melanoma patients who did not respond to immunotherapy exerted increased cellular senescence in their CD8+ T-cells, CD4+ T-cells, B-cells (CD19+/CD20+) and NK cells compared to responders. High senescence levels in non-responders were independent of patients' age and not an outcome of immunotherapy, in contrast to conventional anti-cancer treatments. Overall, our findings support cellular senescence of immune cells within the tumor microenvironment, as a potent determinant of the response to immunotherapy.

## Supplementary Information

Supplementary Material 1. Fig S1: The senescence molecular signature, SeneVick. Pipeline followed for SeneVick extraction (a), SeneVick’s genes directionality and composition (b) and how these genes are clustered in one pie chart (left panel) and the manner in which the same genes (right panel) are distributed across major functional categories identified through GO enrichment and biochemical pathway analysis. (c) Genes were grouped into representative clusters including cell cycle regulation, DNA damage response, chromatin remodeling, inflammatory signaling, metabolic processes, and secretory phenotype regulation. Categories were defined based on overrepresented GO terms and curated pathway annotations from KEGG, Reactome, and GeneCards. The chart highlights the relative abundance of genes involved in each biological process, reflecting the multifaceted molecular landscape of cellular senescence. (d) Venn diagram showing overlapping genes across SeneVick and the two most robust senescence signatures is depicted. *The *CDKN2A* gene (p16^INK4A^) is commonly unattainable to be detected in high throughput data due to several technical factors. Bulk RNA-seq might not capture low-expressing transcripts and if p16^INK4A^ transcripts are degraded into fragments, short-read RNA-seq might fail to capture full-length sequences [93].

Supplementary Material 2. Figure S2: SeneVick efficiently detects senescence across tissues and upon age. Violin Plots demonstrate significant SeneVick enrichment in different cell types (a) and upon time (b) in aged vs young mice (n=19 male and n=11 female, GSE132042). Two data sets were compared with the Wilcoxon test, *P < 0.05, **P < 0.01, ***P < 0.001, ****P < 0.0001

Supplementary Material 3. Figure S3: SeneVick exerts increased sensitivity and specificity in demarcating senescent cells from non-senescent ones compared to other senescence detecting signatures. a. Right panel: Indication of the different timepoints on UMAP plot of the scRNA data from human fibroblasts in the GSE226225 dataset. b. UMAP plot categorizing cells based on SeneVick, SenMayo and FridMan enrichment upon time (days) in the GSE226225 dataset showing the scRNA data of human fibroblasts (GSE226225) representing the enrichment of the three signatures upon time following treatment. c. Timepoint analysis (*P*<0,05) showing the gradual enrichment of the signatures across days following etoposide treatment from the scRNA data of GSE226225 and demonstrating the occurring breakpoints (day 1). The enrichment levels of SeneVick (top), SenMayo (middle) and FridMan (bottom) in human fibroblasts, in which the induction of senescence was accomplished with different senescent inducers (Irradiation-IR and ETO) were compared to proliferative fibroblasts. Significance was assessed by Wilcoxon Test, *P*< 2.22e-16. *P < 0.05, **P < 0.01, ***P < 0.001, ****P < 0.0001

Supplementary Material 4. Figure S4: Workflow for the integration of scRNA patients’ data from the two melanoma studies. Schematic illustration of data preprocessing (Box 1), quality control (Box 2), normalization (Box 3), and cell annotation for the integration of the scRNA data of the Rs and NRs melanoma patients following immunotherapy from the GSE115978 (*n*=30) and GSE120575 (*n*=18) datasets.

Supplementary Material 5. Figure S5: Gating strategy followed for the identification of peripheral blood mononuclear cell (PBMC) subpopulations and the assessment of GLF16+ (senescent) cells. Exclusion of acquisition artefacts was performed by plotting events over time. Single cells were selected by sequential gating on FSC-A/FSC-H and SSC-A/SSC-H dot plots. Live cells were identified with BD Horizon™ Fixable Viability Stain 570. In a CD14/CD16 plot, monocytes were gated and further classified into classical (CD14++CD16-), intermediate (CD14++CD16+), and non-classical (CD14+CD16++). CD14-cells were plotted in a CD3/CD19 plot to identify B-cells, further classified in a CD27/CD38 plot to plasma cells (CD27+CD38+), naïve B-cells (CD27-CD38-) and memory B-cells (CD27+CD38-). In the same plot, gated CD3-CD19 negative cells were shown in a CD16/CD56 plot, and gated NK cells were further classified as CD56bright, CD56+ and CD56dim. Finally, T/NKT cells were distinguished in a CD56/SSC-A plot, and T-cells were further shown in a CD4/SSC-A plot to gate CD8+ and CD4+ T-cells. Tregs were identified as CD4+CD127-CD25high. CD4+, CD8+ T and NKT cells were subdivided in CD27/CD28 plots. Percentages of GLF16+ cells in CD4+ and CD8+ T-cells, NK and B-cells are shown as inserts

Supplementary Material 6. Figure S6: GLF16+ (senescent) cell % percentages of immune subsets of Rs and NRs melanoma patients. a. Workflow depicting cellular senescence assessment in circulating CD4+ and CD8+ T-cells and B-cells (CD19+/CD20+) from melanoma patients by applying the senescence detecting algorithm (SDA). b-c. Representative dot plots from 4 melanoma patients, showing the percentages of GLF16+ cells (in insets) in CD4+ and CD8+ T-cells, and B-cells (CD19+/CD20+) isolated from two Rs (Responder 1 and 2) (b) and two NRs (Non-Responder 1 and 2) (c). Human and vessel icons were provided by Servier Medical Art (https://smart.servier.com/), licensed under CC BY 4.0 (https://creativecommons.org/licenses/by/4.0/).”

Supplementary Material 7. Figure S7: Serial section analysis reveals increased immune cell senescence in the TME of NRs compared to Rs to immunotherapy. Representative images of serial section analysis in melanoma lesions stained with the senescence detecting reagent GL13 and markers for CD4+ and CD8+ T-cells, and B-cells. Rs (upper panel) depict lower senescence in CD4, CD8 and CD20 cells within the TME compared to NRs (lower panel: insets and red arrows) Objective 10× (1^st^ and 3^rd^ line), 40× (2^nd^ and 4^th^). Scale bars: 30 μm and 60 μm, respectively. Cell icons were provided by Servier Medical Art (https://smart.servier.com/), licensed under CC BY 4.0 (https://creativecommons.org/licenses/by/4.0/).”

Supplementary Material 8. Figure S8: Ligand-Receptor interactions among the secreting and recipient cells of Rs and NRs patients. a. Left column: Pie charts illustrating the secreting cells for each ligand receptor interaction of Rs patients which promote effective immune responses that are absent in NRs patients. Middle column: Pie charts depicting the recipient cells with their receptor for each ligand receptor interaction of Rs which promote effective immune responses that are absent in NRs patients. Right column: The result of each ligand receptor interaction between the secreting and the recipient cells and the immune function that is being regulated. b. Left column: Pie charts illustrating the secreting cells for each ligand receptor interaction of NRs patients which promote ineffective immune responses that are absent in Rs patients. Middle column: Pie charts depicting the recipient cells with their receptor for each ligand receptor interaction of NRs which promote ineffective immune responses that are absent in Rs patients. Right column: The result of each ligand receptor interaction between the secreting and the recipient cells and the immune function that is being deregulated

Supplementary file 9. Figure S9: Overview of cancer driven mechanisms inducing immune cell senescence in the TME. Schematic illustration of putative mechanisms involved in cancer promoted immune cell senescence in the tumor microenvironment. Due to cancer cell’s accelerated growth and metabolism the TME is characterized by hypoxia, high levels of reactive oxygen (ROS) and nitrogen (RNS) species and lipids that solely or in concert induce DNA damage response, eventually triggering immune cell senescence [94]. Interestingly, tumor cells have been reported to induce senescence in T-cells by c-AMP delivery or by transferring mitochondria with mtDNA mutations [95, 96]. Moreover, tumor-derived immunoglobulin-like transcript 4 (ILT4), an inhibitory molecule of the immunoglobulin superfamily, has been shown to induce T-cell senescence via activation of ERK1/2 MAPK signaling [97]. Tumor-associated Tregs can also induce T-cell senescence in responding naïve/effector T-cells, by promoting mitochondrial disruption and p38/ERK1/2 MAPK signaling activation as well as via increased glucose consumption and metabolic competition [98].

Supplementary Material 10

Supplementary Material 11

Supplementary Material 12

Supplementary Material 13

Supplementary Material 14

## Data Availability

RNA-seq data obtained from 7-, 35-,75- years old primary human fibroblasts and from senescent and non-senescent PBMCs, processed values and complete gene lists are openly available at Zenodo (doi:10.5281/zenodo.17587925).

## Abbreviations

SA-β-Gal: Senescence-associated β-galactosidase
SDA: Senescence detecting algorithm
FFPE: Formalin-fixed and paraffin-embedded
GCA: Giant cell arteritis
SASP: Senescence-associated secretory phenotype
scRNA: Single cell ribonucleic acid sequencing
Rs: Responders
NRs: Non-Responders
TME: Tumor microenvironment
NK: Natural killer
H202: Hydrogen Peroxide
GEO: Gene expression omnibus
IR: Irradiation
ETO: Etoposide
DMEM: Dulbecco's modified eagle medium
FBS: Fetal bovine serum
PBMCs: Human peripheral blood mononuclear cells
RPMI: Roswell park memorial institute medium
PBS: Phosphate-buffered saline
ROS: Reactive oxygen species
GO: Gene ontology
BP: Biological process
MF: Molecular function
CC: Cellular component
DAVID: Database for annotation, visualization and integrated discovery
FDR: False discovery rate
KEGG: Kyoto encyclopedia of genes and genomes
DNA: Deoxyribonucleic acid
DDR: DNA damage response
PCA: Principal component analysis
PFA: Paraformaldehyde
DMSO: Dimethyl sulfoxide
RT: Room temperature
IgG: Ιmmunoglobulin G
H&L: Heavy and light chains
ICC: Immunocytochemistry
DAB: 3,3′-Diaminobenzidine
T: Telomere
S: Signal
PCR: Polymerase chain reaction
QC: Quality control
MBG: Molecular biology grade water
Alb: Αlbumin
PNA-FISH: Peptide nucleic acid fluorescence in situ hybridization
RNase: Ribonuclease
TBS: Tris-buffered saline
HCL: Hydrochloric acid
SSC: Saline sodium citrate
SDS: Sodium dodecyl sulfate
STAR: Spliced transcripts alignment to a reference
GSEA: Gene set enrichment analysis
MTS: 3-(4,5-Dimethylthiazol-2-yl)-5-(3- carboxymethoxyphenyl)-2-(4-sulfophenyl)-2H-tetrazolium
TPM: Transcripts per million
RECIST: Response evaluation criteria in solid tumours
ROC: Receiver operating characteristic
AUC: Area under the curve
BOR: Best overall response
FACS: Fluorescence-activated cell sorting
DPBS: Dulbecco’s phosphate-buffered saline
SPSS: Statistical package for social sciences
IBM: International business machines
NES: Normalized enrichment score
pRB: Retinoblastoma protein
MAPK: Mitogen-activated protein kinase
cAMP: Cyclic adenosine monophosphate
ICI: Immune checkpoint inhibition
UMAP: Uniform manifold approximation and projection
LR: Ligand-receptor
RNS: Reactive nitrogen species
