# Supplementary figures and images for: Immune cell senescence drives responsiveness to immunotherapy in melanoma
Source: Mol Cancer. 2025 Dec 10;24:308. doi: 10.1186/s12943-025-02517-1 (PMC12717699; doi:10.1186/s12943-025-02517-1)

Figure S1

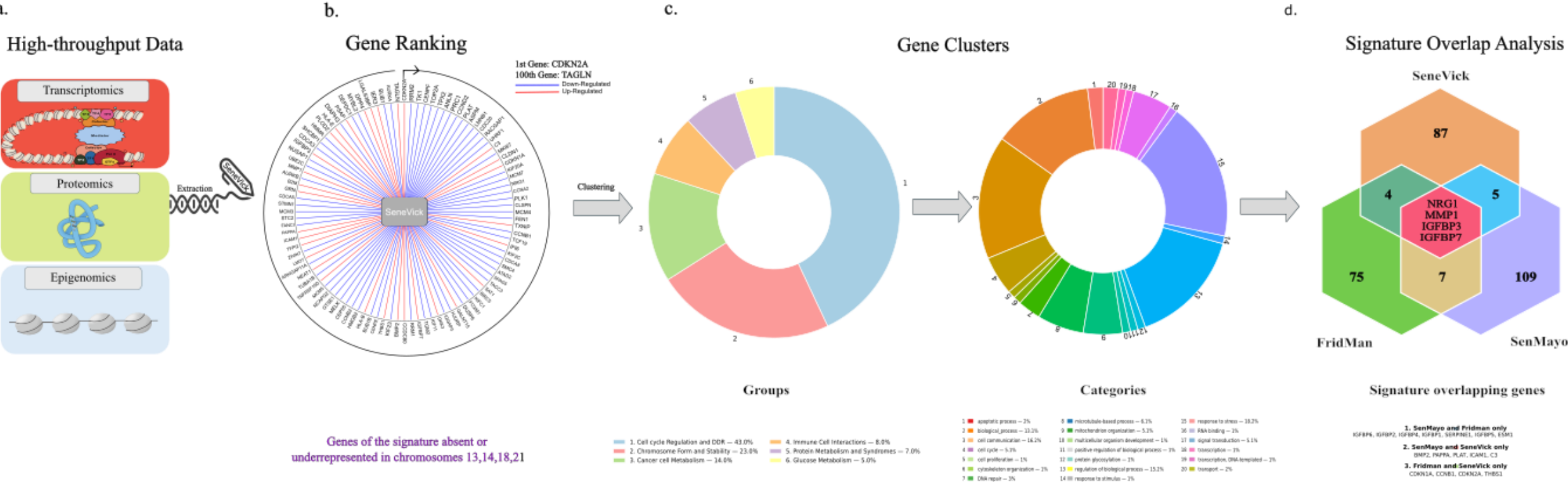

Supplement: Supplementary file 1 — Supplementary Material 1. Fig S1: The senescence molecular signature, SeneVick. Pipeline followed for SeneVick extraction (a), SeneVick’s genes directionality and composition (b) and how these genes are clustered in one pie chart (left panel) and the manner in which the same genes (right panel) are distributed across major functional categories identified through GO enrichment and biochemical pathway analysis. (c) Genes were grouped into representative clusters including cell cycle regulation, DNA damage response, chromatin remodeling, inflammatory signaling, metabolic processes, and secretory phenotype regulation. Categories were defined based on overrepresented GO terms and curated pathway annotations from KEGG, Reactome, and GeneCards. The chart highlights the relative abundance of genes involved in each biological process, reflecting the multifaceted molecular landscape of cellular senescence. (d) Venn diagram showing overlapping genes across SeneVick and the two most robust senescence signatures is depicted. *The CDKN2A gene (p16INK4A) is commonly unattainable to be detected in high throughput data due to several technical factors. Bulk RNA-seq might not capture low-expressing transcripts and if p16INK4A transcripts are degraded into fragments, short-read RNA-seq might fail to capture full-length sequences [93]. [file 12943_2025_2517_MOESM1_ESM.pdf]

Figure S2

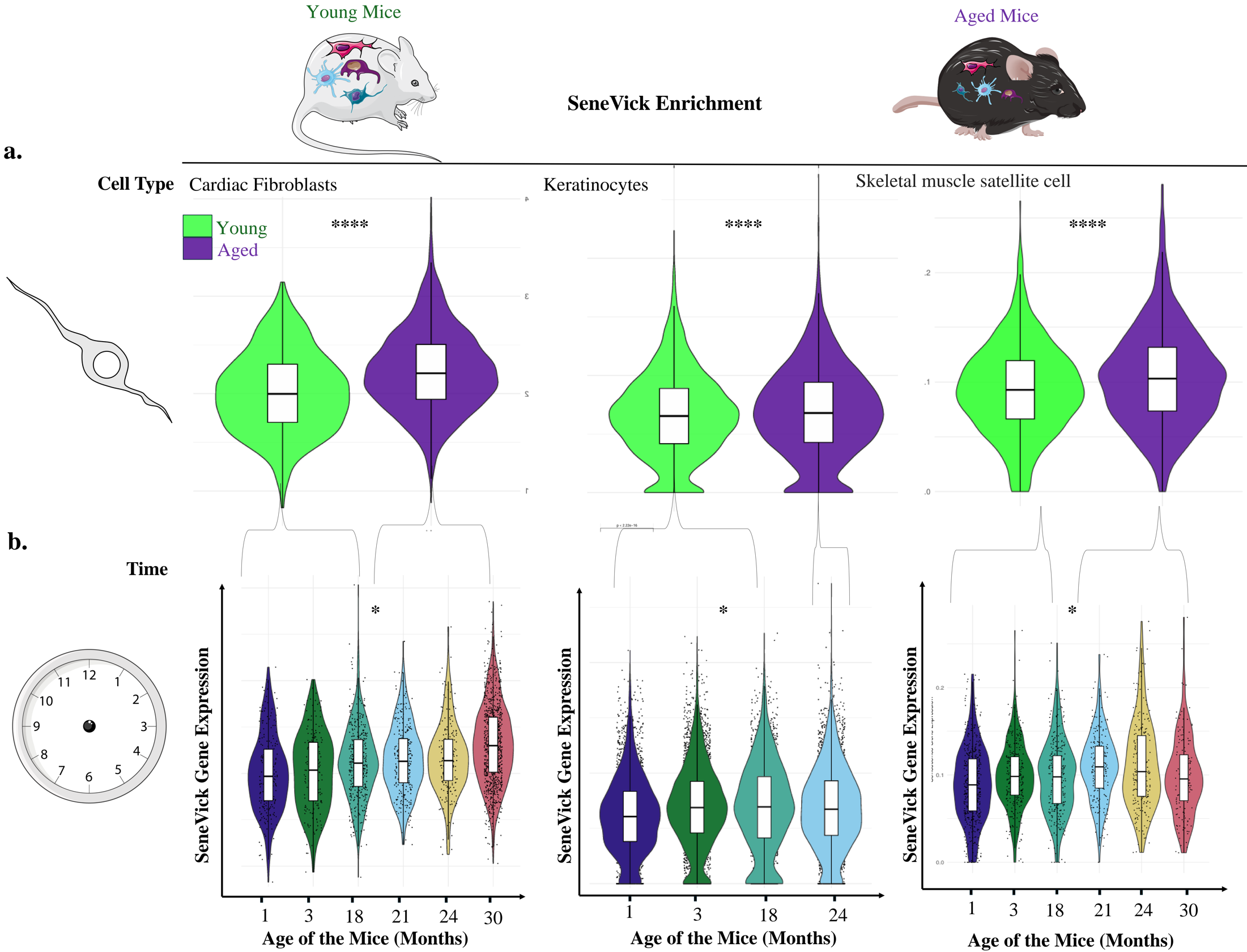

Supplement: Supplementary file 2 — Supplementary Material 2. Figure S2: SeneVick efficiently detects senescence across tissues and upon age. Violin Plots demonstrate significant SeneVick enrichment in different cell types (a) and upon time (b) in aged vs young mice (n=19 male and n=11 female, GSE132042). Two data sets were compared with the Wilcoxon test, *P < 0.05, **P < 0.01, ***P < 0.001, ****P < 0.0001 [file 12943_2025_2517_MOESM2_ESM.pdf]

Figure S4

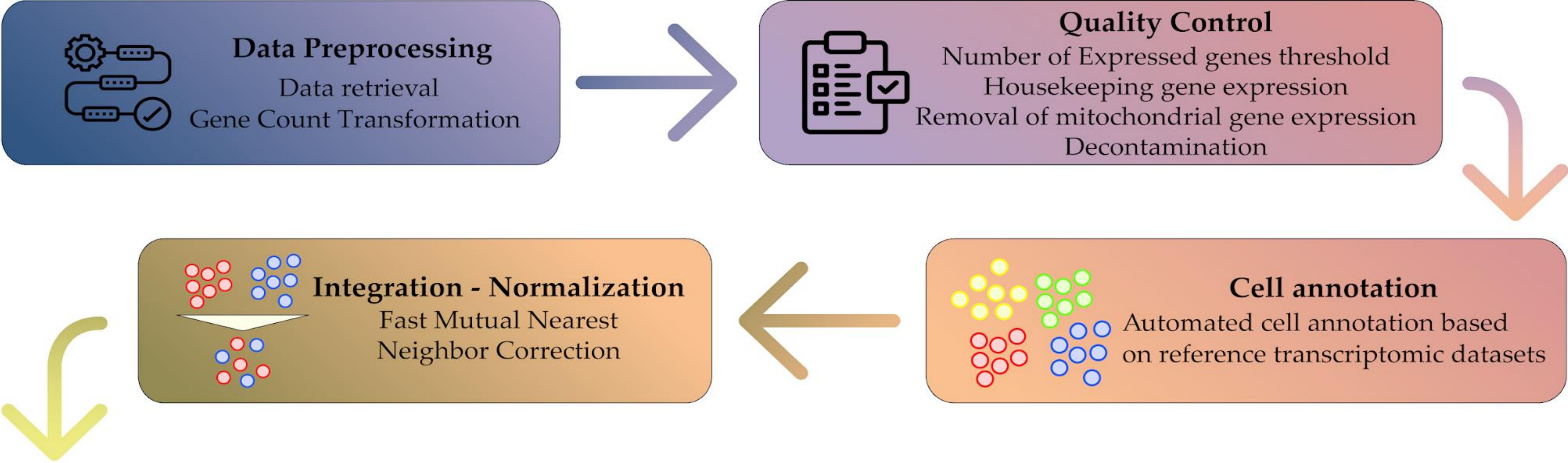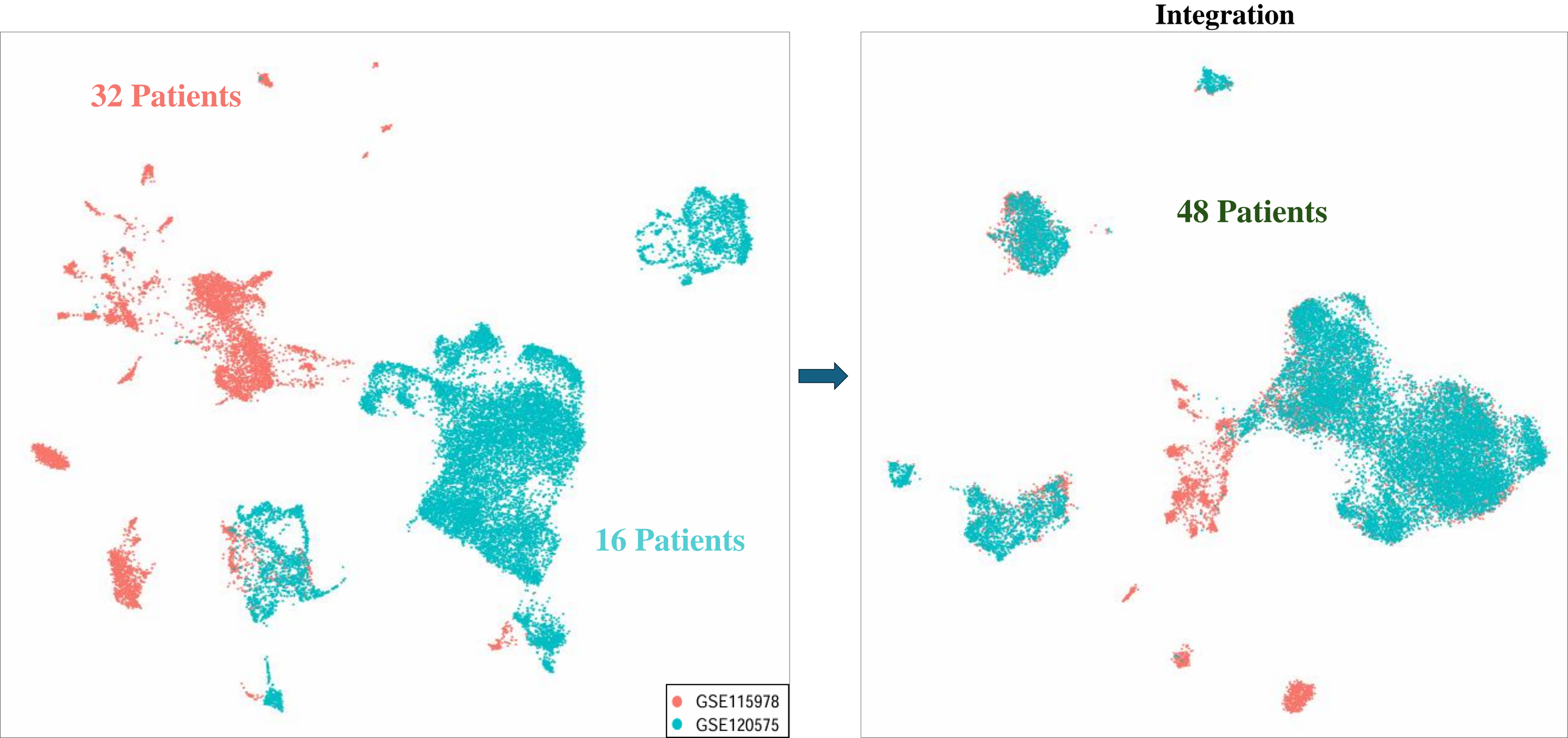

Supplement: Supplementary file 4 — Supplementary Material 4. Figure S4: Workflow for the integration of scRNA patients’ data from the two melanoma studies. Schematic illustration of data preprocessing (Box 1), quality control (Box 2), normalization (Box 3), and cell annotation for the integration of the scRNA data of the Rs and NRs melanoma patients following immunotherapy from the GSE115978 (n=30) and GSE120575 (n=18) datasets. [file 12943_2025_2517_MOESM4_ESM.pdf]

Figure S5

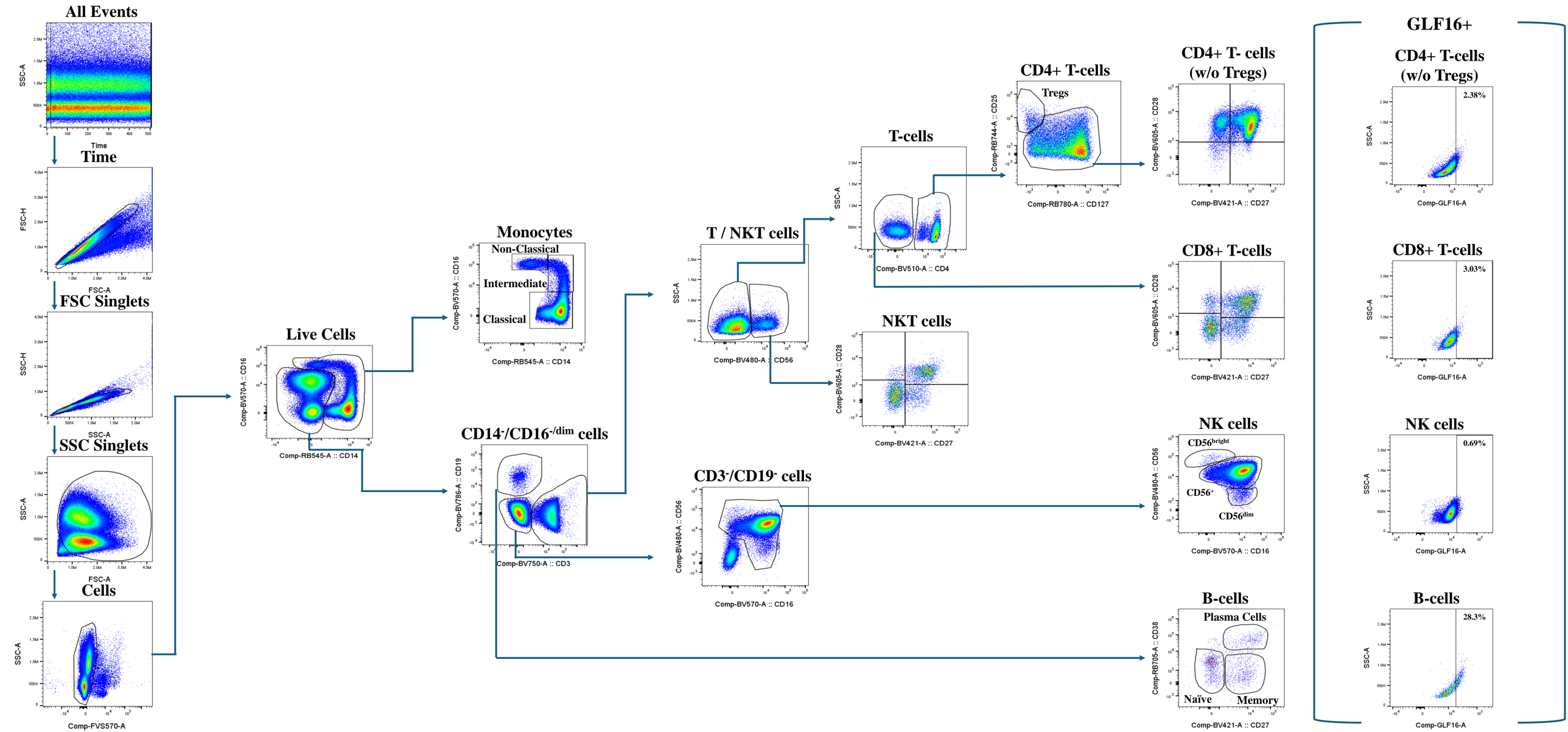

Supplement: Supplementary file 5 — Supplementary Material 5. Figure S5: Gating strategy followed for the identification of peripheral blood mononuclear cell (PBMC) subpopulations and the assessment of GLF16+ (senescent) cells. Exclusion of acquisition artefacts was performed by plotting events over time. Single cells were selected by sequential gating on FSC-A/FSC-H and SSC-A/SSC-H dot plots. Live cells were identified with BD Horizon™ Fixable Viability Stain 570. In a CD14/CD16 plot, monocytes were gated and further classified into classical (CD14++CD16-), intermediate (CD14++CD16+), and non-classical (CD14+CD16++). CD14-cells were plotted in a CD3/CD19 plot to identify B-cells, further classified in a CD27/CD38 plot to plasma cells (CD27+CD38+), naïve B-cells (CD27-CD38-) and memory B-cells (CD27+CD38-). In the same plot, gated CD3-CD19 negative cells were shown in a CD16/CD56 plot, and gated NK cells were further classified as CD56bright, CD56+ and CD56dim. Finally, T/NKT cells were distinguished in a CD56/SSC-A plot, and T-cells were further shown in a CD4/SSC-A plot to gate CD8+ and CD4+ T-cells. Tregs were identified as CD4+CD127-CD25high. CD4+, CD8+ T and NKT cells were subdivided in CD27/CD28 plots. Percentages of GLF16+ cells in CD4+ and CD8+ T-cells, NK and B-cells are shown as inserts [file 12943_2025_2517_MOESM5_ESM.pdf]

**Figure S6**

**a.**

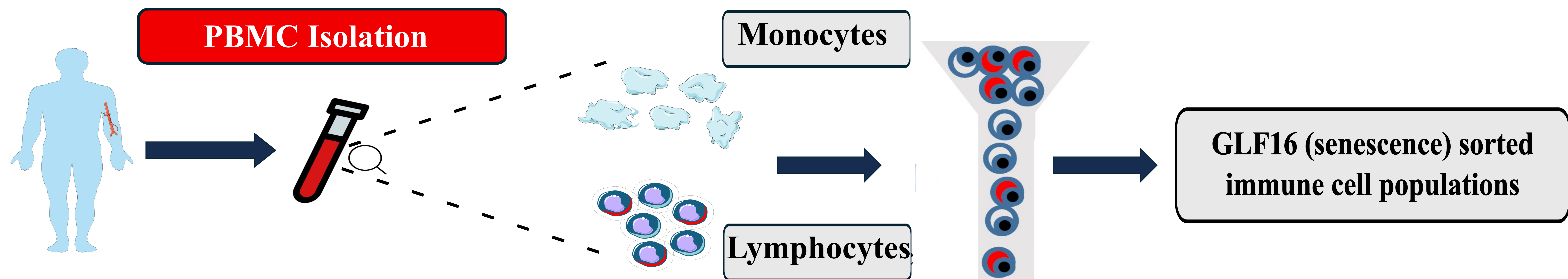

**b.**

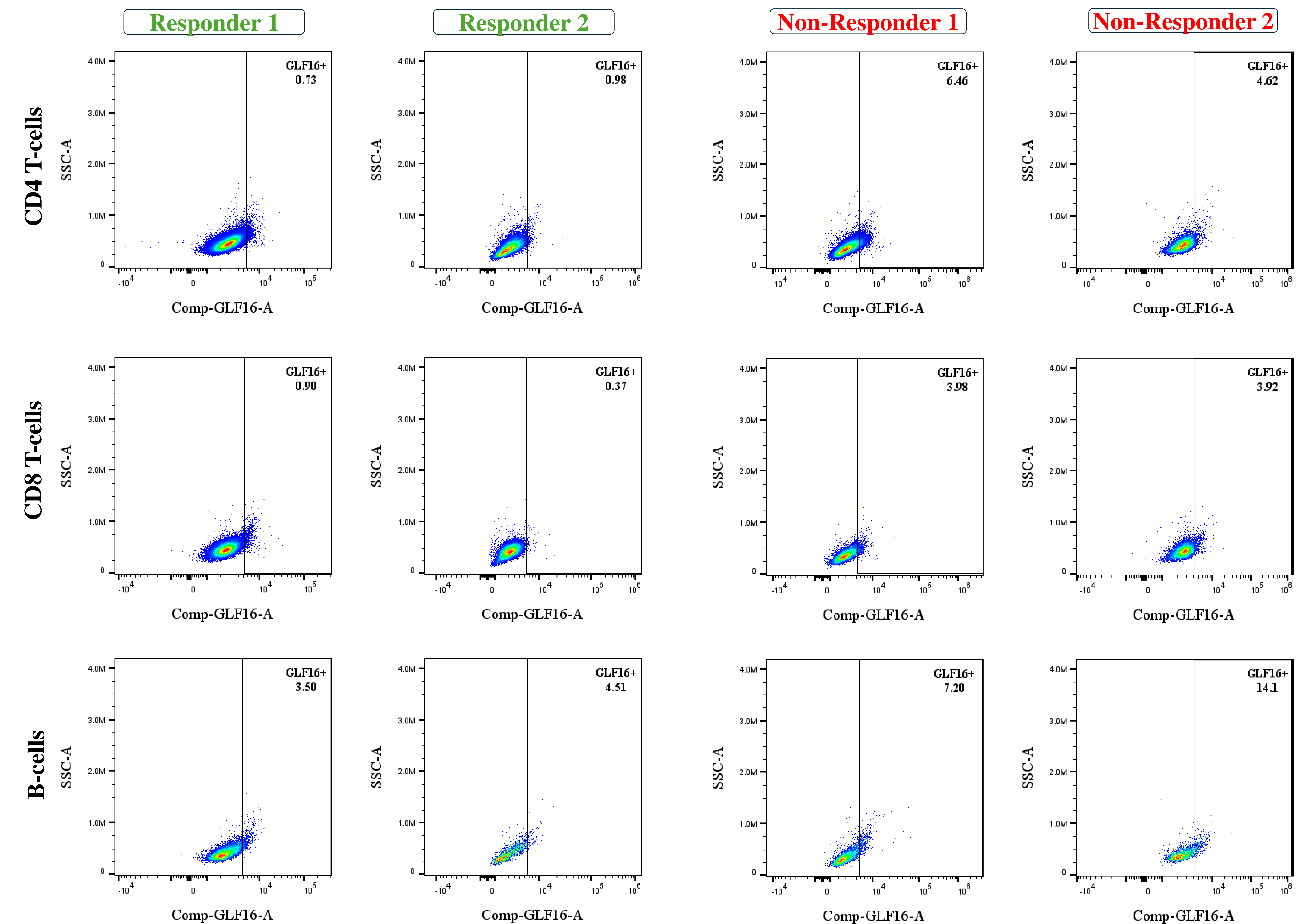

**c.**

Supplement: Supplementary file 6 — Supplementary Material 6. Figure S6: GLF16+ (senescent) cell % percentages of immune subsets of Rs and NRs melanoma patients. a. Workflow depicting cellular senescence assessment in circulating CD4+ and CD8+ T-cells and B-cells (CD19+/CD20+) from melanoma patients by applying the senescence detecting algorithm (SDA). b-c. Representative dot plots from 4 melanoma patients, showing the percentages of GLF16+ cells (in insets) in CD4+ and CD8+ T-cells, and B-cells (CD19+/CD20+) isolated from two Rs (Responder 1 and 2) (b) and two NRs (Non-Responder 1 and 2) (c). Human and vessel icons were provided by Servier Medical Art (https://smart.servier.com/), licensed under CC BY 4.0 (https://creativecommons.org/licenses/by/4.0/).” [file 12943_2025_2517_MOESM6_ESM.pdf]

**Figure S7**

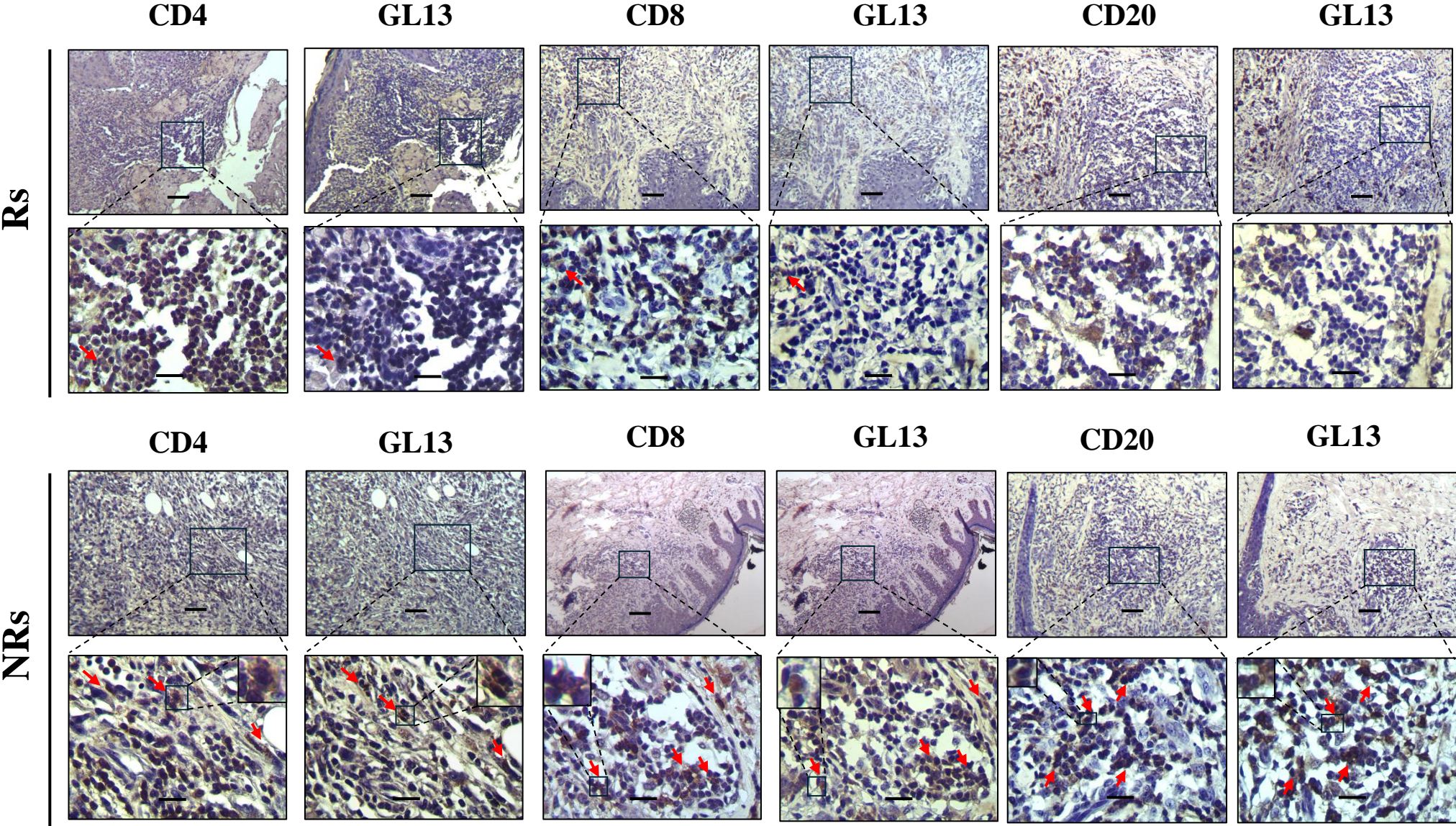

Supplement: Supplementary file 7 — Supplementary Material 7. Figure S7: Serial section analysis reveals increased immune cell senescence in the TME of NRs compared to Rs to immunotherapy. Representative images of serial section analysis in melanoma lesions stained with the senescence detecting reagent GL13 and markers for CD4+ and CD8+ T-cells, and B-cells. Rs (upper panel) depict lower senescence in CD4, CD8 and CD20 cells within the TME compared to NRs (lower panel: insets and red arrows) Objective 10× (1st and 3rd line), 40× (2nd and 4th). Scale bars: 30 μm and 60 μm, respectively. Cell icons were provided by Servier Medical Art (https://smart.servier.com/), licensed under CC BY 4.0 (https://creativecommons.org/licenses/by/4.0/).” [file 12943_2025_2517_MOESM7_ESM.pdf]

Figure S9

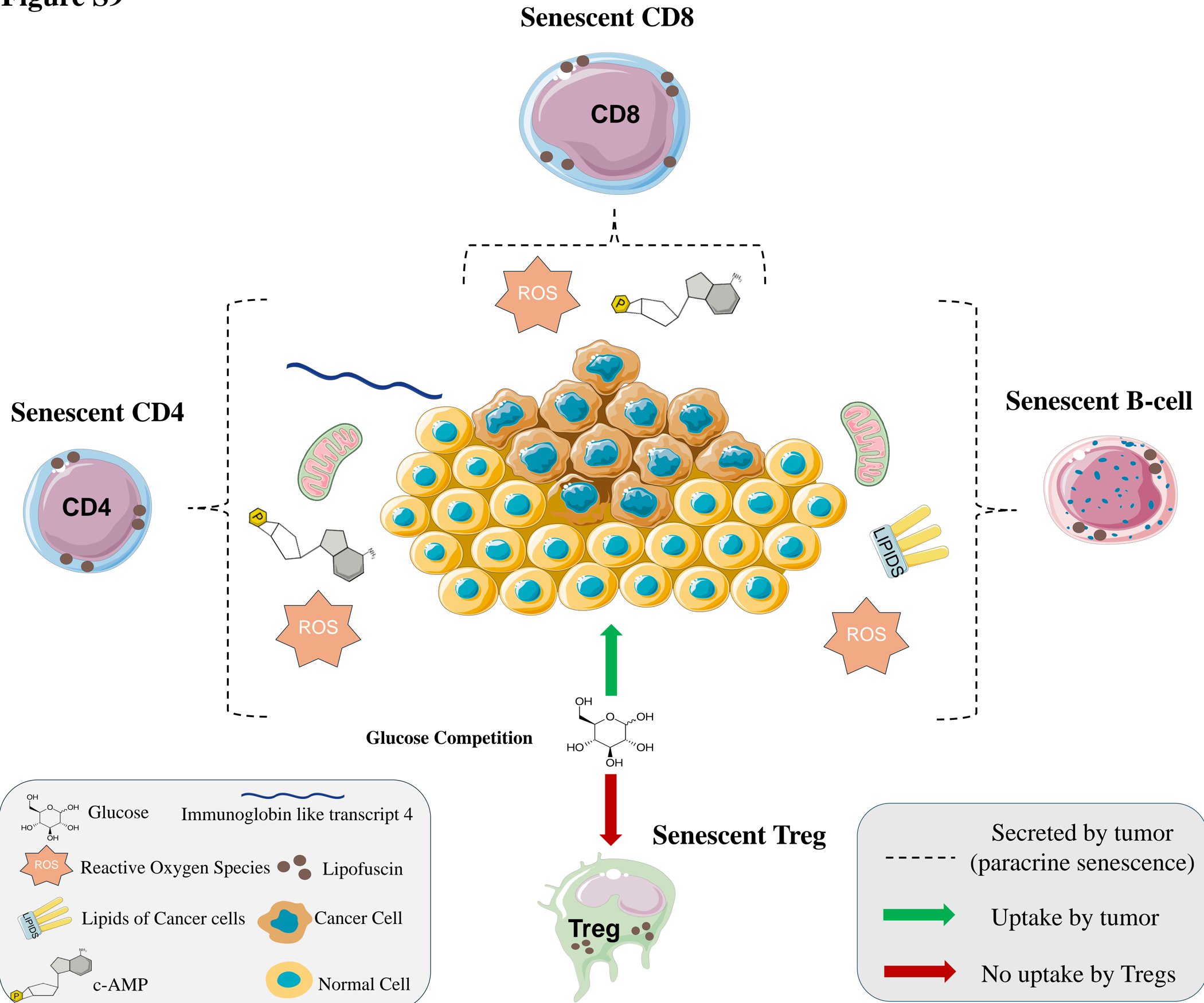

Supplement: Supplementary file 9 — Figure S9: Overview of cancer driven mechanisms inducing immune cell senescence in the TME. Schematic illustration of putative mechanisms involved in cancer promoted immune cell senescence in the tumor microenvironment. Due to cancer cell’s accelerated growth and metabolism the TME is characterized by hypoxia, high levels of reactive oxygen (ROS) and nitrogen (RNS) species and lipids that solely or in concert induce DNA damage response, eventually triggering immune cell senescence [94]. Interestingly, tumor cells have been reported to induce senescence in T-cells by c-AMP delivery or by transferring mitochondria with mtDNA mutations [95, 96]. Moreover, tumor-derived immunoglobulin-like transcript 4 (ILT4), an inhibitory molecule of the immunoglobulin superfamily, has been shown to induce T-cell senescence via activation of ERK1/2 MAPK signaling [97]. Tumor-associated Tregs can also induce T-cell senescence in responding naïve/effector T-cells, by promoting mitochondrial disruption and p38/ERK1/2 MAPK signaling activation as well as via increased glucose consumption and metabolic competition [98]. [file 12943_2025_2517_MOESM9_ESM.pdf]
