# Supplementary material for: Immune cell senescence drives responsiveness to immunotherapy in melanoma
Source: Mol Cancer. 2025 Dec 10;24:308. doi: 10.1186/s12943-025-02517-1 (PMC12717699; doi:10.1186/s12943-025-02517-1)
Supplement: Supplementary file 3 — Supplementary Material 3. Figure S3: SeneVick exerts increased sensitivity and specificity in demarcating senescent cells from non-senescent ones compared to other senescence detecting signatures. a. Right panel: Indication of the different timepoints on UMAP plot of the scRNA data from human fibroblasts in the GSE226225 dataset. b. UMAP plot categorizing cells based on SeneVick, SenMayo and FridMan enrichment upon time (days) in the GSE226225 dataset showing the scRNA data of human fibroblasts (GSE226225) representing the enrichment of the three signatures upon time following treatment. c. Timepoint analysis (P<0,05) showing the gradual enrichment of the signatures across days following etoposide treatment from the scRNA data of GSE226225 and demonstrating the occurring breakpoints (day 1). The enrichment levels of SeneVick (top), SenMayo (middle) and FridMan (bottom) in human fibroblasts, in which the induction of senescence was accomplished with different senescent inducers (Irradiation-IR and ETO) were compared to proliferative fibroblasts. Significance was assessed by Wilcoxon Test, P< 2.22e-16. *P < 0.05, **P < 0.01, ***P < 0.001, ****P < 0.0001 [file 12943_2025_2517_MOESM3_ESM.pdf]

Figure S3

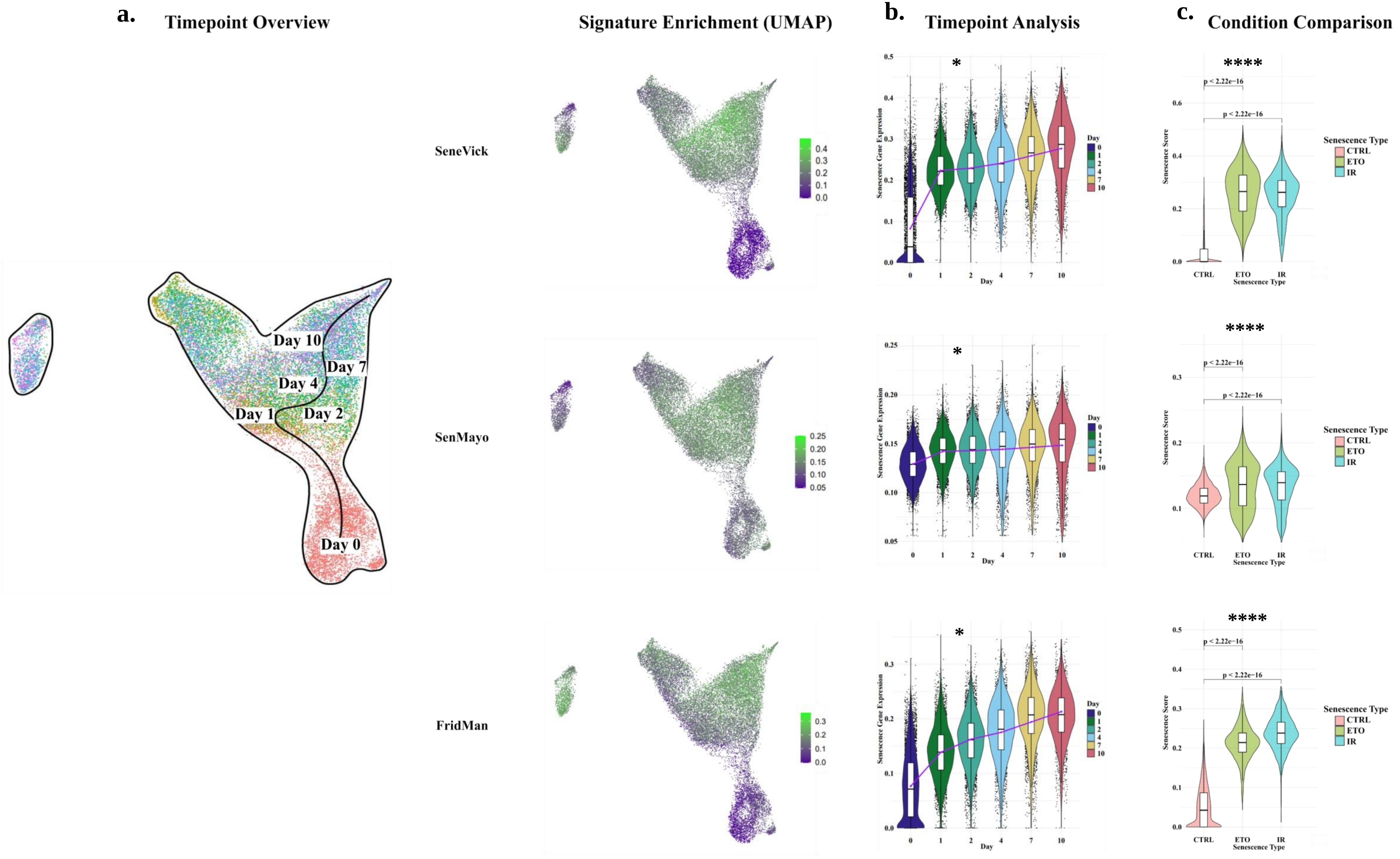

a.
Timepoint Overview
Signature Enrichment (UMAP)
b.
Timepoint Analysis
c.
Condition Comparison
Day 10
Day 7
Day 4
Day 1
Day 2
Day 0
SeneVick
SenMayo
FridMan
Senescence Gene Expression
Day
Senescence Score
Senescence Type
CTRL
ETO
IR
p < 2.22e−16
*
****
