# Supplementary material for: Immune cell senescence drives responsiveness to immunotherapy in melanoma
Source: Mol Cancer. 2025 Dec 10;24:308. doi: 10.1186/s12943-025-02517-1 (PMC12717699; doi:10.1186/s12943-025-02517-1)
Supplement: Supplementary file 8 — Supplementary Material 8. Figure S8: Ligand-Receptor interactions among the secreting and recipient cells of Rs and NRs patients. a. Left column: Pie charts illustrating the secreting cells for each ligand receptor interaction of Rs patients which promote effective immune responses that are absent in NRs patients. Middle column: Pie charts depicting the recipient cells with their receptor for each ligand receptor interaction of Rs which promote effective immune responses that are absent in NRs patients. Right column: The result of each ligand receptor interaction between the secreting and the recipient cells and the immune function that is being regulated. b. Left column: Pie charts illustrating the secreting cells for each ligand receptor interaction of NRs patients which promote ineffective immune responses that are absent in Rs patients. Middle column: Pie charts depicting the recipient cells with their receptor for each ligand receptor interaction of NRs which promote ineffective immune responses that are absent in Rs patients. Right column: The result of each ligand receptor interaction between the secreting and the recipient cells and the immune function that is being deregulated [file 12943_2025_2517_MOESM8_ESM.pdf]

Figure S8

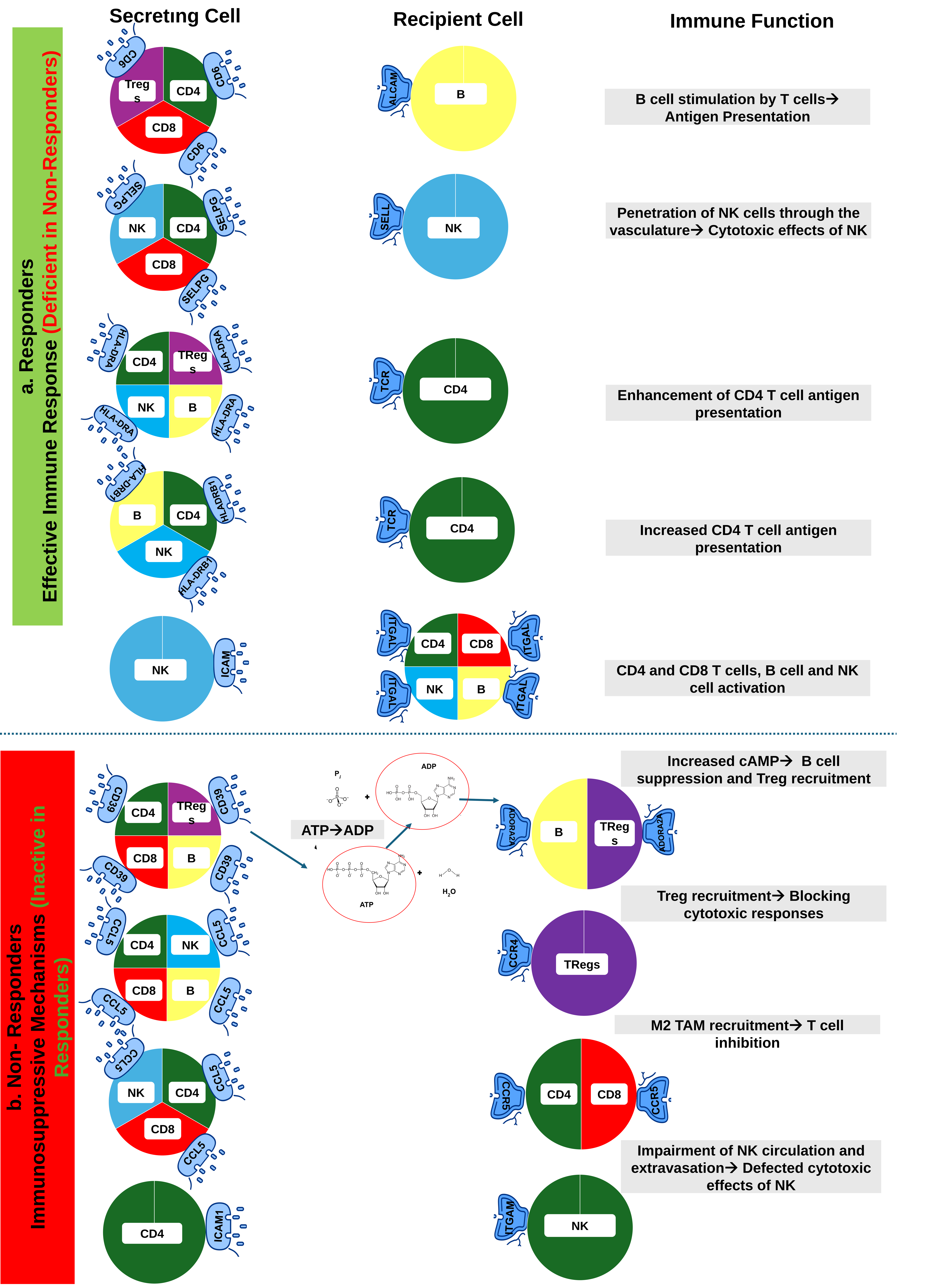
Secreting Cell
Recipient Cell
Immune Function
a. Responders
Effective Immune Response (Deficient in Non-Responders)
Treg s
CD4
CD8
CD6
ALCAM
B
B cell stimulation by T cells→ Antigen Presentation
NK
SELPG
SELL
Penetration of NK cells through the vasculature→ Cytotoxic effects of NK
TReg s
HLA-DRA
TCR
Enhancement of CD4 T cell antigen presentation
HLA-DRB1
HLADRB1
Increased CD4 T cell antigen presentation
ICAM
ITGAL
CD4 and CD8 T cells, B cell and NK cell activation
b. Non- Responders
Immunosuppressive Mechanisms (Inactive in Responders)
CD39
ATP→ADP
ADP
ATP
H_2O
Increased cAMP→ B cell suppression and Treg recruitment
ADORA2A
Treg recruitment→ Blocking cytotoxic responses
CCR4
TRegs
CCL5
M2 TAM recruitment→ T cell inhibition
CCR5
ICAM1
ITGAM
Impairment of NK circulation and extravasation→ Defected cytotoxic effects of NK
